# Integrative analyses of genetic variation in enzyme activities of primary carbohydrate metabolism reveal distinct modes of regulation in *Arabidopsis thaliana*

**DOI:** 10.1186/gb-2008-9-8-r129

**Published:** 2008-08-18

**Authors:** Joost JB Keurentjes, Ronan Sulpice, Yves Gibon, Marie-Caroline Steinhauser, Jingyuan Fu, Maarten Koornneef, Mark Stitt, Dick Vreugdenhil

**Affiliations:** 1 Laboratory of Genetics, Wageningen University, Arboretumlaan, NL-6703 BD Wageningen, The Netherlands; 2 Laboratory of Plant Physiology, Wageningen University, Arboretumlaan, NL-6703 BD Wageningen, The Netherlands; 3 Centre for Biosystems Genomics, Droevendaalsesteeg, NL-6708 PB Wageningen, The Netherlands; 4 Max Planck Institute for Molecular Plant Physiology, Am Mühlenberg, 14476 Potsdam-Golm, Germany; 5 Groningen Bioinformatics Centre, Groningen Biomolecular Sciences and Biotechnology Institute, University of Groningen, Kerklaan, NL-9751 NN Haren, The Netherlands; 6 Max Planck Institute for Plant Breeding Research, Carl-von-Linné-Weg 10, 50829, Cologne, Germany

## Abstract

**Background:**

Plant primary carbohydrate metabolism is complex and flexible, and is regulated at many levels. Changes of transcript levels do not always lead to changes in enzyme activities, and these do not always affect metabolite levels and fluxes. To analyze interactions between these three levels of function, we have performed parallel genetic analyses of 15 enzyme activities involved in primary carbohydrate metabolism, transcript levels for their encoding structural genes, and a set of relevant metabolites. Quantitative analyses of each trait were performed in the *Arabidopsis thaliana *L*er *× Cvi recombinant inbred line (RIL) population and subjected to correlation and quantitative trait locus (QTL) analysis.

**Results:**

Traits affecting primary metabolism were often correlated, possibly due to developmental control affecting multiple genes, enzymes, or metabolites. Moreover, the activity QTLs of several enzymes co-localized with the expression QTLs (eQTLs) of their structural genes, or with metabolite accumulation QTLs of their substrates or products. In addition, many trait-specific QTLs were identified, revealing that there is also specific regulation of individual metabolic traits. Regulation of enzyme activities often occurred through multiple loci, involving both *cis*- and *trans*-acting transcriptional or post-transcriptional control of structural genes, as well as independently of the structural genes.

**Conclusion:**

Future studies of the regulatory processes in primary carbohydrate metabolism will benefit from an integrative genetic analysis of gene transcription, enzyme activity, and metabolite content. The multiparallel QTL analyses of the various interconnected transducers of biological information flow, described here for the first time, can assist in determining the causes and consequences of genetic regulation at different levels of complex biological systems.

## Abstract 2

Multiparallel QTL analysis of 15 *Arabidopsis* primary carbohydrate metabolism enzymes reveals that traits affecting primary metabolism are often correlated.

## Background

Carbon is probably the most prevalent and important element in any life form. Whereas most other organisms are dependent on intake of organic forms of carbon, plants fix inorganic carbon through photosynthesis. Upon fixation, most of the inorganic carbon is converted into sucrose, which in most plants acts as the major source of organic carbon for further metabolism. Some of the fixed carbon is temporarily stored as starch, and remobilized at night to support respiration or used for continued sucrose synthesis and export to other tissues. To meet the various demands of a growing plant for specific purposes, carbohydrates need to be allocated within the plant, and converted into a plethora of compounds [1].

Carbohydrate metabolism is more complex in plants than in most other organisms. For example, there are alternative routes for the mobilization and metabolization of diverse components [2]. Depending on the tissue, part or all of the glycolytic pathway is present in the plastid as well as in the cytosol [3]. As a result, a given substrate may be converted into different products, and products can be formed from different substrates. In addition, most enzymes in plant central metabolism are encoded by small gene families [4,5]. This versatility enables different metabolic routes and creates a dense metabolic network with short pathway lengths. Perturbations in sub-parts of the network can have strong consequences for other parts and ultimately may affect plant growth and development [6-8]. The complexity of the metabolic network allows the plant to compensate for disturbance in one route by enhancing flux through an alternative route [9]. To ensure a balanced carbon allocation through a plant's life-cycle, a strong and tight regulation is essential. At the same time, this complexity means that there may be considerable redundancy, at least under standardized growth conditions. Indeed, there are several reports where major changes in the expression of individual enzymes lead to little change in metabolism (for example, [10-12]).

Given the huge diversity in plant species, with large differences in their energy metabolism, growth and storage of reserves, it can be expected that there will be considerable variation in primary carbohydrate metabolism between species, and most likely also within species. Large differences have been observed in many enzyme activities and metabolite contents in *Arabidopsis*, between accessions [13,14], and depending on the growing conditions [15-17], developmental stages [18], time of day [19], and tissues [20,21]. For a thorough understanding of the role of natural variation in plant primary metabolism and development it is of pivotal importance to identify the genetic basis of variation in metabolic pathways and processes.

The study of natural variation in primary metabolism might also contribute more generally to our understanding of the integration of metabolism with growth. In a recent study 24 *Arabidopsis *accessions were analyzed for biomass production, metabolite content, and enzyme activity [13]. Significant correlations were observed between biomass, enzyme activities, and carbohydrates. Further evidence for connectivity between plant development and primary metabolism is derived from other studies [18,22]. Here gas chromatography-mass spectrometry metabolic profiling of the Col × C24 recombinant inbred line (RIL) and near isogenic line population was used in parallel with biomass determinations. Although there were no strong correlations between individual metabolites and biomass production, a strong canonical correlation was observed when all metabolites were taken into account. Among the metabolites contributing most to the observed correlation were intermediates of the hexose phosphate pool: fructose-6-phosphate, α-D-glucose-6-phosphate (G6P), and α-D-glucose-1-phosphate (G1P). While occasionally positive correlations between biomass and metabolites were observed, the large majority of metabolites, including sucrose, hexose phosphates and members of the tricarboxylic acid cycle, showed negative correlations. These studies indicate that high rates of biomass production and increased fluxes as a result of higher enzyme activities lead to depletion of the pools of metabolites. A similar conclusion was reached by studying the relationship between tomato fruit size and metabolite content [23]. Natural variation in, and spatial and temporal control of, primary carbohydrate metabolism, therefore, suggest a tight relationship with plant development, although it is difficult to assess cause and consequence and this regulation might be highly complex.

Natural variation can be effectively analyzed in mapping populations, offering the possibility of locating genetic factors that are causal for the observed variation [24]. RIL populations offer unique possibilities for such integrative studies because different types of experiments can be performed in replicates on the same genotypes. Furthermore, a large number of genetic perturbations segregate in populations derived from crosses of distinct accessions. Depending on the population size, a relatively large set of lines can then be analyzed for correlations between traits, as well as the genetic regulation of these traits via identification of quantitative trait loci (QTLs) controlling variation observed for these traits. The advantage of *Arabidopsis *is that its genome has been sequenced [4] and genes have been (putatively) annotated for nearly all enzymes in primary metabolism [25], allowing analysis of transcriptional regulation of these genes.

Genetics has already been successfully used to analyze quantitative variation in central plant metabolism [14,20-23,26-34]. However, most studies addressed only a limited number of enzymes or metabolites. While others have combined information on transcript levels and metabolites [35], none have integrated information across all three levels or incorporated quantitative genetic variation. Genetic studies benefit enormously from multidisciplinary approaches [36-38]. To gain insight into connectivity in metabolic networks it is therefore recommendable to analyze as many enzymes and metabolites involved in such a network as possible, and to combine these with a parallel analysis of gene expression [15,35,39-41].

In the present study, we analyzed the activity of 15 different enzymes involved in primary carbohydrate metabolism and compared this with information about the transcript levels for their structural genes and the levels of the most important carbohydrates and related metabolites in the Landsberg *erecta *(L*er*) × Cape verde islands (Cvi) RIL population of *Arabidopsis thaliana *[42]. Although this population is of moderate size, we show that genetically controlled variation exists for the activity of many enzymes as well as for transcript levels of their structural genes and for the metabolites they interconvert. By comparing the localization and responses of structural genes encoding the enzymes with expression QTLs (eQTLs) for their transcript levels, and QTLs for enzyme activities and metabolite contents, we demonstrate that genetically controlled regulation occurs through different modes of action and at multiple levels.

## Results

### Natural variation in primary carbohydrate metabolism

**Figure 1:**
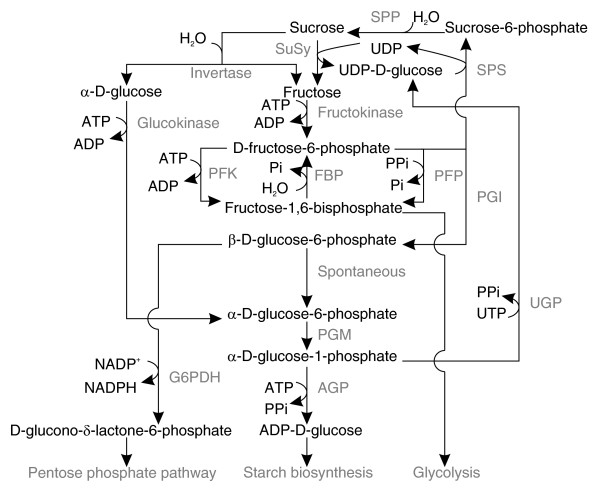
Enzymatic conversions in primary carbohydrate metabolism. Reactions are given in the biologically most relevant direction, although several enzymes can catalyze reversible reactions. Metabolites are depicted in black and converting enzymes are depicted in gray. SPP, sucrose-phosphate phosphatase.

To determine the extent of natural variation in primary carbohydrate metabolism in *Arabidopsis *we analyzed a RIL population derived from a cross between the two distinct accessions L*er *and Cvi [42]. Metabolic conversion rates attributable to enzyme activity were established for 15 specific enzymatic reactions, in parallel with determinations of pools of selected metabolites (Table 1, Figure 1). The enzyme assays were performed in optimized conditions to measure maximum velocity (Vmax) activities, which should be proportional to the level of protein [15,40]. The metabolites measured included structural components (total protein, chlorophyll), major products of photosynthesis (starch, sucrose, reducing sugars, total amino acids), and short-lived intermediates in the pathways of carbohydrate synthesis (G6P, G1P, UDP-D-glucose (UDPG)).

**Table 1:** Summation of enzymes and metabolites analyzed

Trait	Full name	Reaction
INV	Acid soluble invertase, vacuolar	*Sucrose + H_2_O *→ *α-D-glucose + fructose*
AGP	ADP-glucose pyrophosphorylase	*ADP-D-glucose + PPi *→ *α-D-glucose-1-phosphate + ATP*
FBP	Fructose-1,6-bisphosphate phosphatase, cytosolic isoform	*Fructose-1,6-bisphosphate + H_2_O *→ *D-fructose-6-phosphate + Pi*
G6PDH	Glucose-6-phosphate 1-dehydrogenase	*β-D-glucose-6-phosphate + NADP^+ ^*→ *D-glucono-δ-lactone-6-phosphate + NADPH*
PFK	ATP dependent phosphofructokinase	*D-fructose-6-phosphate + ATP *→ *fructose-1,6-bisphosphate + ADP*
PFP	Pyrophosphate: fructose-6-phosphate 1-phosphotransferase	*D-fructose-6-phosphate + PPi *→ *fructose-1,6-bisphosphate + Pi*
PGM	Phosphoglucomutase	*α-D-glucose-1-phosphate *→ *α-D-glucose-6-phosphate*
PGI	Phosphoglucose isomerase, cytosolic and plastidial isoforms	*D-fructose-6-phosphate *→ *β-D-glucose-6-phosphate*
SPS	Sucrose phosphate synthase	*D-fructose-6-phosphate + UDP-D-glucose *→ *sucrose-6-phosphate + UDP*
SuSy	Sucrose synthase	*Sucrose + UDP *→ *UDP-D-glucose + fructose*
GK	Glucokinase	*α-D-glucose + ATP *→ *α-D-glucose-6-phosphate + ADP*
FK	Fructokinase	*Fructose + ATP *→ *D-fructose-6-phosphate + ADP*
UGP	UDP-glucose pyrophosphorylase	*UDP-D-glucose + PPi *→ *α-D-glucose-1-phosphate + UTP*
Rubisco	Ribulose bisphosphate carboxylase/oxygenase, initial and upon maximum activation	*H_2_O + CO_2 _+ D-ribulose-1,5-bisphosphate *→ *2 3-phosphoglycerate + 2 H^+^*
		
Protein	Total protein content	
ChlA	Chlorophyl A	
ChlB	Chlorophyl B	
		
AA	Total amino acids	
Starch	Starch	
Suc	Sucrose	
Glu	Glucose	
Fru	Fructose	
G1P	α-D-glucose-1-phosphate	
G6P	α-D-glucose-6-phosphate	
UDPG	UDP-D-glucose	

Reactions are given in the direction as they were assayed although several enzymes can also catalyze the reversible reactions.

Considerable variation was observed within the population for most of the analyzed traits, with heritability estimates up to 90% (phosphoglucomutase (PGM); Table 2), indicating that a substantial part of the observed variation could be attributed to genetic factors, as was also concluded from QTL analyses. Heritability was below 20% for INV (acid soluble invertase, vacuolar), plastid phosphoglucose isomerase (cytosolic and plastidial isoforms; PGI) and Ribulose bisphosphate carboxylase/oxygenase (Rubisco) activities and, in general, less QTLs were detected for low heritability traits.

**Table 2:** Genetic analyses of analyzed traits

						Log_2 _(A/B)	
						
Trait	H^2^	Chr.	Mb	LOD	%Expl. Var	QTL	Parents
Inv	0.19	1	4.1	5.3	13.7	-0.32	-0.13
AGP	0.42	4	12.4	3.1	8.0	0.19	-0.02
FBP	0.26	5	14.0	3.5	9.6	-0.33	-1.00
G6PDH	0.37						-0.94
PFK	0.33						-0.38
PFP	0.70						0.36
PGM	0.90	1	26.9	16.0	17.5	0.42	-0.37
		5	20.9	36.4	56.3	-0.78	
PGI(Cyt)	0.71	1	16.8	3.1	6.8	0.18	0.35
		2	11.2	5.4	12.7	0.24	
		5	17.2	4.0	8.9	0.22	
PGI(Pla)	0.11	5	16.7	3.1	8.4	-0.20	0.33
PGI(Tot)	0.06	1	14.9	3.2	8.8	0.13	0.34
SPS	0.41	5	7.0	6.4	18.0	0.27	0.36
SuSy	0.25						0.07
GK	0.28						ND
FK	0.21	5	16.6	3.6	9.4	-0.44	ND
UGP	0.51	3	0.8	17.1	37.8	-0.40	0.12
		5	5.2	5.1	9.3	0.20	
Rubisco (Ini)	0.16						0.16
Rubisco (Max)	0.23	3	20.5	3.1	9.0	0.19	0.21
Rubisco (Ratio)	0.08						-0.50
							
Protein	0.81	2	12.9	3.2	7.6	0.16	0.35
		3	7.4	3.2	7.6	0.12	
ChlA	0.63	2	11.2	3.7	7.4	0.11	0.43
		3	0.3	3.4	6.8	0.11	
		4	10.6	3.4	6.7	0.11	
		5	1.7	3.8	7.6	0.12	
ChlB	0.37						0.32
							
AA	0.62	2	8.5	5.3	8.9	-0.14	-0.53
		2	16.2	3.9	6.2	-0.12	
		3	0.3	4.7	7.5	0.12	
		4	13.9	5.1	8.6	-0.13	
		5	14.0	4.1	6.6	-0.11	
Starch	0.45						-0.04
Suc	0.34	3	15.6	3.4	8.5	-0.13	0.39
		3	23.3	5.8	15.1	0.17	
Glu	0.70	1	4.9	8.5	19.2	-0.28	0.10
		2	11.2	4.4	9.1	-0.18	
		3	13.0	5.8	13.8	-0.27	
Fru	0.49	1	5.4	5.0	10.9	-0.20	0.03
		3	7.9	11.7	27.5	0.34	
		3	13.0	6.2	15.3	-0.28	
							
G1P	0.47	3	0.3	4.5	12.1	-0.33	-0.56
		5	7.2	3.3	8.8	0.28	
G6P	0.39	3	1.3	4.0	13.0	-0.37	-0.38
UDPG	0.43	3	0.8	35.9	64.9	-0.58	-0.71
							
PC1		2	11.2	4.7	11.6	1.05	
PC2		3	0.3	28.2	54.6	-2.48	
PC3		1	4.4	4.7	13.0	-1.17	
PC4							
PC5		5	8.6	4.1	11.9	-1.00	
PC6		3	7.0	7.1	19.0	1.87	
PC7		5	18.2	10.8	28.5	-1.56	
PC8		5	1.3	4.2	11.9	1.21	

The second to eighth columns represent, respectively, the heritability for trait values within the RIL population (H^2^), the chromosome number on which a QTL was detected (Chr.), the position of the QTL on the chromosome in Mbp (Mb), the LOD score, percentage of the total variance explained (%Expl. Var) and effect of the QTL and the parental genotype on trait values (Log_2 _A/B; A = L*er*, B = Cvi). A principle components analysis was also performed (PC1-8, principal components 1-8; for more details see Table 3).

### Identification of QTLs involved in primary metabolism

**Figure 2:**
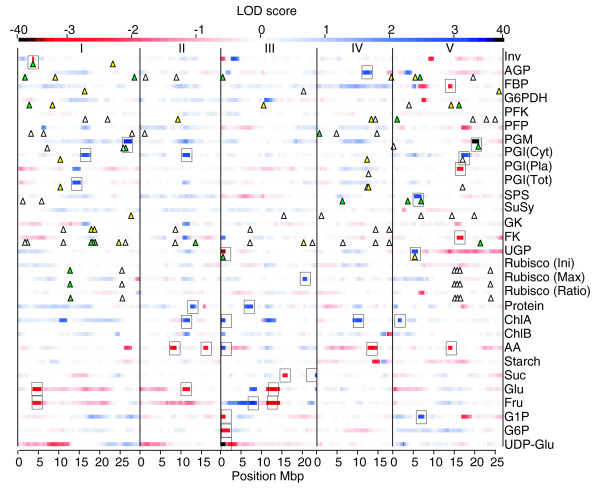
Heatmap of QTL profiles of each analyzed trait. Color intensities represent LOD scores. Positive effect loci are projected in blue and negative effect loci in red. Significantly detected QTLs are boxed. Chromosomal borders are indicated by vertical shaded lines and the position of structural genes for the enzyme by triangles. Transcriptional regulation of structural genes is indicated by different colors of the triangles: green, local eQTL; yellow, distant eQTL; white, no eQTLs detected or gene not analyzed. AA, total amino acids; Cyt, cytosolic; Ini, initial; Max, maximum; Pla, plastidial; Tot, total.

**Figure 3:**
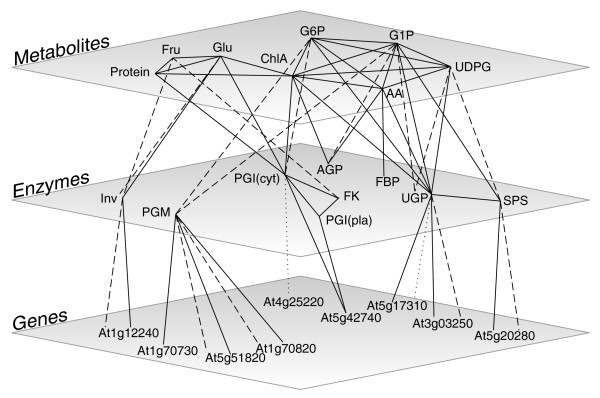
QTL co-location network of analyzed genes, enzymes and metabolites. Edges between genes and enzymes represent: solid, position of structural gene co-locating with enzyme activity QTL; dashed, *cis*-eQTL co-locating with enzyme activity QTL; dotted, *trans*-eQTL co-locating with enzyme activity QTL. Edges between enzymes and metabolites represent: solid, enzyme activity QTL co-locating with metabolite content QTL; dashed, enzymes connected to their substrate and/or product metabolites. Solid edges within planes connect traits with co-locating QTLs. Co-location was defined as an overlap in 2 Mbp QTL support intervals. AA, total amino acids; cyt, cytosolic; pla, plastidial.

Significant QTLs were detected for 10 out of 15 of the enzyme activity traits and 9 out of 11 of the metabolite level traits (Table 2, Figure 2). In general, the overall effect of QTLs for a given trait was in concordance with the phenotypic differences observed between the parents. Multiple QTLs were detected for several traits, sometimes with opposite effects. This could contribute to the large variation and transgression that was observed. The data were analyzed for co-location of QTLs, defined as an overlap in 2 Mbp support intervals (Table 2, Figures 2 and 3). Few co-locating QTLs were detected for the different enzyme activities, even though several of the enzymes are from the same or related pathways (Table 2, Figures 1 and 3). Co-location was more frequent for metabolite content QTLs. This may be partly because more QTLs were detected for metabolite levels than for enzyme activities. The detection of many trait-specific QTLs indicates that there is strong and independent genetic regulation of the metabolic traits investigated in this study.

### Correlations between metabolic traits across the RIL population

**Figure 4:**
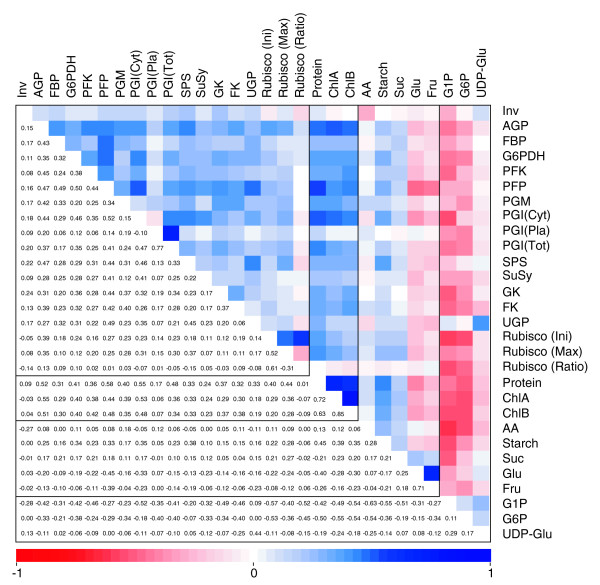
Correlation matrix of analyzed enzymes and metabolites. Values and shading intensities represent spearman rank correlation coefficients between two traits. Values in bold face are significant at a Bonferroni corrected *p*-value of 1.00E-5. AA, total amino acids; Cyt, cytosolic; Ini, initial; Max, maximum; Pla, plastidial; Tot, total.

Despite this independent genetic regulation, many of the metabolic traits correlated with each other across the RIL population. For example, there is a tight correlation between chlorophyl A (ChlA) and chlorophyl B (ChlB). While several QTLs were found for ChlA, only suggestive QTLs were found for ChlB at similar positions (Figure 2). Likewise, plastidic PGI contributes to total PGI activity but QTLs were found on different positions for both traits. Suggestive QTLs were again found at identical positions. A positive correlation was also found between the activities of most of the enzymes (Figure 4). There was also a positive correlation between many enzyme activities and the structural metabolites protein and chlorophyll. A weaker positive correlation was observed between many enzyme activities and sucrose, amino acids, and starch, and a weak negative correlation with reducing sugars. This group of metabolites represents the end products of photosynthesis, and the primary compounds resulting from nitrogen incorporation. They are exported to other parts of the plant or, in the case of starch, temporarily stored in the leaf and remobilized for export in the night. Stronger negative correlations were observed between enzyme activities and intermediates of metabolic pathways, such as G1P, G6P, and UDPG. Taken together, these findings suggest that higher enzyme activities may allow higher fluxes, while lowering the levels of the intermediary substrates in the pathways. Occasional exceptions (for example, between UDP-glucose pyrophosphorylase (UGP) and UDPG) will be discussed later.

### Principle components analysis

To determine a possible common factor that explains the observed correlations, we performed a principal component analysis on all traits analyzed. For most traits, a large part of the variation could be extracted in eight principal components (PCs), which together explained 68% of the observed variation (Table 3). By far the most representative was PC1, which explained over 28% of the variance. Interestingly, in PC1, positive values were obtained for the enzyme activity traits and some metabolite end products, while negative values were obtained for hexose levels. This is in line with the observed correlations between these traits (see above).

**Table 3:** Principal component analysis

	Total	PC1	PC2	PC3	PC4	PC5	PC6	PC7	PC8
Inv	0.44	0.22	0.27	0.41	-0.24	0.06	0.26	-0.17	-0.01
AGP	0.64	0.78	0.06	0.10	-0.05	0.09	-0.01	0.03	0.05
FBP	0.53	0.48	0.21	0.15	-0.04	0.17	0.22	0.31	-0.24
G6PDH	0.59	0.70	-0.11	0.09	-0.06	0.23	0.09	-0.14	-0.10
PFK	0.42	0.56	0.02	-0.04	0.04	0.00	0.28	-0.15	-0.08
PFP	0.82	0.83	0.21	-0.06	-0.11	0.03	-0.05	0.13	-0.21
PGM	0.65	0.54	0.04	0.15	-0.19	0.09	-0.02	0.49	0.20
PGI(Cyt)	0.70	0.76	0.12	-0.09	0.01	-0.08	0.13	-0.25	-0.10
PGI(Pla)	0.84	0.33	-0.11	0.30	-0.54	-0.19	-0.54	-0.06	0.01
PGI(Tot)	0.89	0.71	-0.04	0.16	-0.38	-0.22	-0.34	-0.23	-0.01
SPS	0.58	0.65	0.30	0.11	0.07	-0.15	-0.13	-0.04	-0.06
SuSy	0.35	0.45	0.14	-0.01	-0.12	-0.01	0.15	-0.05	-0.30
GK	0.51	0.60	0.02	0.02	-0.31	0.04	0.17	-0.11	0.06
FK	0.54	0.49	-0.23	0.06	-0.28	0.15	0.17	0.31	0.13
UGP	0.72	0.51	0.57	0.16	0.25	0.05	-0.18	0.08	-0.04
Rubisco (Ini)	0.91	0.51	-0.20	0.10	0.33	0.53	-0.41	-0.16	0.13
Rubisco (Max)	0.73	0.54	0.01	0.07	0.40	-0.29	-0.37	-0.10	0.21
Rubisco (Ratio)	0.93	0.09	-0.24	0.05	0.02	0.91	-0.10	-0.10	-0.03
ChlA	0.83	0.73	-0.24	-0.14	0.20	-0.15	0.25	-0.02	0.32
ChlB	0.78	0.68	-0.19	-0.17	0.11	-0.05	0.36	-0.06	0.33
AA	0.70	0.13	-0.52	-0.01	0.13	-0.08	-0.25	0.51	-0.26
Protein	0.74	0.80	-0.13	-0.10	0.14	-0.10	0.06	0.02	0.18
Starch	0.59	0.55	-0.25	0.02	0.31	-0.18	-0.10	0.15	-0.25
Suc	0.70	0.24	-0.23	0.50	0.48	-0.11	0.19	0.02	-0.24
Glu	0.86	-0.39	-0.30	0.78	0.01	-0.11	0.06	-0.03	0.00
Fru	0.79	-0.27	-0.39	0.68	-0.01	-0.03	0.22	-0.07	0.23
G1P	0.69	-0.14	0.57	0.13	-0.01	0.04	0.00	0.39	0.43
G6P	0.48	-0.16	0.54	0.22	0.09	0.02	0.23	0.01	-0.23
UDPG	0.70	-0.13	0.69	0.26	0.27	0.09	-0.19	-0.07	0.15
									
% of variance	67.82	28.25	9.08	6.64	5.47	5.36	5.25	4.00	3.77

Columns represent, respectively, the proportion of variance that could be explained by all components and by each component separately for the different traits analyzed. The last row represents the percentage of variance of all traits explained by all the components together, and by each separate component.

When the corresponding PC values for the individual RILs were subjected to QTL analysis, a strong QTL for PC1 was observed at 11.2 Mbp on chromosome 2. This corresponds to the position of the *ERECTA *locus (Table 2; see Discussion for more details). Some traits showed a significant QTL at this position (protein, ChlA, PGI and glucose (Glu)), and several others showed a non-significant suggestive QTL (PGM, glucokinase (GK), fructokinase (FK) and ChlB). Other traits did not show an indication of a QTL at this position, even though PC1 explained a large part of the variation observed for these traits (for example, ADP-glucose pyrophosphorylase (AGP), glucose-6-phosphate 1-dehydrogenase (G6PDH), pyrophosphate:fructose-6-phosphate 1-phosphotransferase (PFP) and sucrose phosphate synthase (SPS)). This might suggest that further loci, which could not significantly be detected, are also involved in the contribution of these traits to PC1. The other PCs accounted for less than 10% of the variance and explain variation in specific subsets of traits. PC2 best explains most of the variation observed for UGP, G1P, G6P and UDPG. All of these traits show a QTL at the same position at the top of chromosome 3 (Table 2), where a QTL for PC2 was also detected (Table 2) (see below for further discussion). PC3 best explains the variation observed for Inv, sucrose (Suc), glucose and fructose (Fru), which, together with PC3, all map at the top of chromosome 1.

### Relationship between structural gene location and enzyme activity QTLs

The structural genes for almost all of the enzymes in primary carbohydrate metabolism have been identified in *Arabidopsis*. As noted in the introduction, in most cases multiple genes have been annotated. This redundancy possibly results from a number of genome duplications during the evolutionary history of *Arabidopsis*, as well as some local tandem duplications [4]. For many, two or more genes are needed to encode enzymes in different subcellular compartments, and more to account for tissue, developmental or environmental differences in activity. However, it should be noted that many of the annotations are based on homology with genes with known biological activity from other organisms, and experimental evidence for biological activity exists for only a limited number of genes. Furthermore, homologous and paralogous genes might have lost or modified their functions, and/or their expression patterns might have changed.

Several cases were found where the position of structural genes co-locates with QTLs for activity of the enzymes that they encode (Figure 2; Table S1 in Additional data file 1). Examples include individual family members for INV, PGI and SPS, and two family members for PGM and UGP. Co-location indicates that the observed variation in enzyme activity may be due to polymorphisms in the encoding structural genes. Polymorphisms could affect: the coding region of genes leading to an alteration of the specific activity or stability of the resulting protein; or promoter regions that affect transcription efficiency and subsequently protein levels. In the former case the changes of activity should be independent of changes of the transcript levels, whereas in the latter case they will be accompanied by qualitatively similar changes of transcript levels.

### Relationship between transcript levels and enzyme activity

To distinguish between these possibilities, we analyzed the transcript levels of all of the putative structural genes. Samples of the biological material that was used to assay the enzyme activities were analyzed on full genome arrays [43]; signal intensities for each RIL were used to calculate the correlation coefficient between individual transcript levels and enzyme activities, and signal ratios of pairs of RILs on the same slide were used for QTL analyses.

In general, there was only a weak to medium correlation between enzyme activities and the transcript levels of the putative structural genes (Table S1 in Additional data file 1; see below for a discussion of possible reasons). However, very strong positive correlations were found for PGM activity/At5g51820 transcript (*p *< E-23), UGP activity/At3g03250 transcript (*p *< E-07) and UGP activity/At5g17310 transcript (*p *< E-06). Further significant positive correlations (*p *< E-04) were found for G6PDH activity/At1g24280 transcript, PFP activity/At1g76550 transcript and PGI activity/At4g25220 transcript and, at a lower significance level (*p *< E-02), for INV activity/At1g12240 transcript, AGP activity/At1g74910 transcript, AGP activity/At5g19220 transcript, ATP dependent phosphofructokinase (PFK) activity/At4g26270 transcript, cytosolic PGI activity/At5g42740 transcript, and SPS activity/At5g20280 transcript). Weak but significant negative correlations were found for AGP activity/At3g03250 transcript and AGP activity/At5g17310 transcript).

Structural genes co-locate with enzyme activity QTLs in the three cases where the activity/transcript correlation was highest (PGM activity/At5g51820 transcript, UGP activity/At3g03259 transcript and UGP activity/At5g17310 transcript), and in some of the cases where the activity/transcript correlations were weaker (INV activity/At1g12240 transcript, cytosolic PGI activity/At5g42740 transcript, SPS activity/At5g20280 transcript). This indicates that part of the variation in enzyme activity can be explained by differential expression of structural genes. This interpretation is further supported by the fact that structural gene transcript levels correlated positively with enzyme activities in almost all of the above examples. The only exception was a small and non-significant negative correlation of PGM activity and At1g70820 transcript (see below for further discussion). Negative correlations could possibly result from temporal shifts in transcription and translation - for example, in genes showing circadian or diurnal rhythms - although other explanations are also possible (see Discussion).

For all enzymes, except UGP (where transcripts of both family members were anyway strongly correlated with enzyme activity), a better correlation was observed between a limited number of individual gene family members than for the family as a whole (Table S1 in Additional data file 1). This might partly be explained by the aforementioned temporal and spatial specificity of gene expression. Including non-additively acting genes in the analysis therefore introduces more noise, masking the effects of informative genes.

### Relationship between eQTLs and enzyme activity

As a next step we subjected the observed transcript levels of the structural genes to QTL analysis. For each encoded enzyme; we found significant QTLs for at least one of the encoding structural genes (eQTLs; Table S1 in Additional data file 1). Some of the eQTLs co-locate with their structural gene (local regulation) and others do not (distant regulation). Locally observed eQTLs indicate that regulation occurs *in cis*, whereas distant eQTLs suggests regulation to occur *in trans *[44].

Examples of strong local regulation of transcription include UGP (At3g03250), PGM (At5g51820), PFK (At5g03300), and hexokinase (At1g50460). As already noted, the transcript levels for several of these genes correlated positively with enzyme activity. Moreover, in many cases there was a co-location between strong local transcriptional regulation of structural genes and a QTL for the activity of the encoded enzyme (for example, UGP (At3g03250), PGM (At1g70820 and At5g51820), SPS (At5g20280), and INV (At1g12240)). These findings again suggest that *cis*-regulatory variation in expression of structural genes contributes to the observed variation in enzyme activity. The only exception was a structural gene for UGP (At5g17310), which showed strong distant transcriptional regulation by a locus close to the structural gene for the other UGP family member.

In other cases, we found significant eQTLs for structural genes that did not co-locate with QTLs for enzyme activity, but for which a significant correlation was observed between the corresponding enzyme activity and the transcript levels of these genes. This is illustrated by cytosolic PGI. A QTL for PGI activity co-locates with a *trans*-acting eQTL (at 11.2 Mbp on chromosome 2) for a PGI structural gene on chromosome 4 (At4g25220) (Table S1 in Additional data file 1). This was the PGI gene family member whose transcripts showed the highest correlation with PGI activity. In such cases, *trans*-acting regulatory variation in structural gene transcription explains observed variation in enzyme activity.

In other cases, the enzyme activity QTL co-located with a structural gene for that enzyme, but no eQTL was found. This is illustrated by PGM and PGI. For each of these enzymes, one of their structural genes co-located with a QTL for the encoding enzyme activity (that is, At1g70730, PGM; At5g42740, cytosolic PGI), but no significant eQTL was observed at this position. This combination indicates that a change in the translation rate of the transcript, the stability of the protein, or the properties of the encoded protein is responsible for the variation in activity.

Finally, cases were found in which significant locally or distantly acting eQTLs for structural genes were detected, without coinciding positions of genes and activity QTLs or co-locating (e)QTLs, and for which there was no significant correlation between transcript level and enzyme activity. These findings might suggest that not all annotated genes actually make a measurable contribution to the observed activity of the putatively encoded enzyme (for possible reasons, see Discussion).

### Relationship between eQTLs and principle components

We also calculated QTLs for PC1-PC8 (Tables 2 and 3), and compared their location and the eQTLs of the structural genes for enzymes (Table S1 in Additional data file 1). Whilst PC1 seems to be independent of variation in structural genes for individual enzymes, most other PCs can be explained by variation in such genes. PC2 maps at the position of At3g03250, a strong *cis*-regulated structural gene encoding UGP; a QTL for PC3 co-locates with a *cis*-regulated gene for INV (At1g12240), PC5 with a *cis*-regulated gene for SPS (At5g20280) and PC7 maps at the position of a *cis*-regulated PGM encoding gene (At5g51820) (Table 2; Table S1 in Additional data file 1). This matches the pattern noted above, in which PC1 captures a set of broad changes in metabolism, and the minor PCs capture more specific sources of genetic variation.

### Epistatic interactions can explain residual variation

Large residual fractions of variance could not be explained by detected QTLs (Table 2). This might reflect the complex genetic regulation of primary carbohydrate metabolism by many QTLs, each with a relatively small effect, which fail to pass the QTL significance threshold. Segregation of these small-effect QTLs would, however, still contribute to transgression and to the large genetic variation that is observed. Moreover, estimates of individual QTL effects can be severely affected by epistatic interactions between loci [45].

To estimate the involvement of epistatic interactions we performed a genome-wide pair-wise analysis of all marker combinations for all traits and genes described in this study. We found evidence for multiple epistatic interactions for all traits and most genes (Table S2 in Additional data file 1). On average, 4.3 epistatic interactions were found per trait ranging from 1 (Glu) to 13 (Rubisco(ratio)). Although some markers are more frequently found in epistatic interaction pairs than others, ranging from 0-15, pairs are scattered over the genome (Figure S1 in Additional data file 2). Nonetheless, several instances were found where traits share epistatic locus pairs. Most prominent is an interaction between the tops of chromosomes 1 and 3, which was detected for several sugars (Suc, G1P and UDPG), structural components (ChlB, protein content, amino acids), the enzyme UGP and enzyme encoding genes (UGP/At3g03250/At5g17310, GK/At1g50460/At4g37840, FK/At1g06020, Sucrose synthase (SuSy)/At3g43190, PGM/At1g70820, AGP/At4g39210 and SPS/At4g10120) (Table S2 in Additional data file 1). Other examples of shared epistatic locus pairs include enzymes involved in the same pathway (SPS/UGP and AGP/PGM), related metabolites (UGP/G1P and ChlA/ChlB) or structural genes and their encoded enzyme activities or products (PGM/At5g51820 and UDPG/At3g03250) (Table S2 in Additional data file 1). This co-location of epistatic loci might well explain why strong correlations between traits do not always coincide with co-locating QTLs, such as was observed for ChlA and ChlB. In addition, for a number of traits epistatically interacting loci were found even though no QTLs could be significantly detected (G6PDH, PFK, PFP, SuSy, GK, ChlB and starch). On the other hand, for a number of traits and genes QTLs were found to be involved in epistatic locus pairs (PGM, protein, G1P, UDPG; At1g30560, At3g03250, At5g03300, At5g17310, At5g51820 and At5g51830) (Table 2; Tables S1 and S2 in Additional data file 1).

In several instances co-location could be observed between structural gene positions and epistatic loci for enzyme activities (AGP/At4g39210, Fructose-1,6-bisphosphate phosphatase, cytosolic isoform (FBP)/At3g54050, G6PDH/At5g35790, PGI/At1g30560, GK and FK/At4g29130, Rubisco/At5g38410, At5g38420 and At5g38430; Tables S1 and S2 in Additional data file 1). For three of these genes (At1g30560, At4g39120 and At5g35790) *trans *eQTL(s) were found, while for the others no significant eQTL could be detected. None of the *trans*-acting eQTLs coincided with the second locus of the epistatic locus pair. Similarly, *cis*-acting epistatic loci affecting gene expression were found at the position of the affected genes (At1g05610, At1g16570, At3g43190, At3g54050, At5g03300, At5g51820, At5g51830, At5g64380). Here, *cis*-acting eQTLs were detected for three genes (At5g03300, At5g51820 and At5g51830) while *trans*-acting eQTLs were detected for another three (At1g05610, At5g51820 and At5g64380). Again, none of the *trans*-acting eQTLs coincided with the second locus of the epistatic locus pair. For those eight genes for which no epistatic interactions could be detected, four did show a QTL (At1g43670, At1g66430, At1g73370 and At3g27300) and the other four did not (At3g20040, At5g37180, At5g48300 and At5g56630).

These results indicate that epistasis contributes heavily to the observed genetic variation and can explain the lack of co-locating QTLs for highly correlated traits.

### Different modes of action in the genetic control of enzymatic activity

The strongest genetically controlled variation was found for PGM and UGP (see above). We therefore investigated the substrate and product levels for these two enzymes. When combined with the parallel analysis of enzyme activity and the transcript levels of the structural genes, this offers the opportunity of gaining deeper insight into the mechanisms of genetic regulation at these specific sites in metabolism.

**Figure 5:**
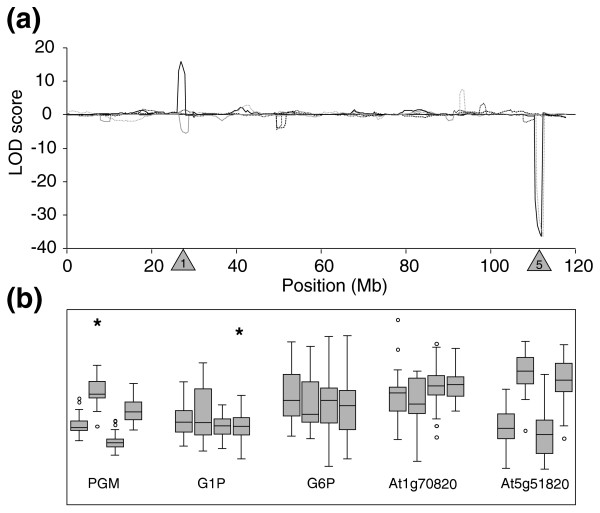
QTL profiles and boxplots of PGM related traits. **(a) **LOD scores plotted against genomic position, the sign of the LOD score is determined by the direction of effect (+, L*er *> Cvi; -, L*er *< Cvi). Black solid line, PGM activity; black dotted line, G1P content; black dashed line, G6P content; gray solid line, At1g70820 expression level; gray dotted line, At5g51820 expression level. Shaded triangles indicate positions of structural genes: 1, At1g70820; 5, At5g51820. **(b) **Boxplots for four genotypic classes. Each class represents genotypic identical individuals for the two QTLs at chromosomes 1 and 5 (from left to right: A_1_A_5_, A_1_B_5_, B_1_A_5_, B_1_B_5_; A = L*er*, B = Cvi). Boxplots show the median, interquartile range, outliers (circles) and extreme cases (asterisks) of individual variables. All traits are plotted in arbitrary units and ranged at similar scale by z-score standardization.

For PGM there are two highly significant activity QTLs, with opposite effects (Figure 5a). The first is a strong activity QTL for PGM at the lower arm of chromosome 5. This activity QTL co-locates with a structural gene for the plastidic PGM (At5g51820; *PGM1*) [46,47] and a strongly significant local eQTL. Lines that contain the L*er *allele at this locus have strongly increased PGM enzyme activity and *PGM1 *transcript levels. The co-location of a structural gene, an activity QTL and an expression QTL and the similar direction of the additive effect of both QTLs strongly suggests that *cis*-regulatory variation in the expression of a structural gene is causal for the observed variation in enzyme activity. The second activity QTL for PGM is located on the lower arm of chromosome 1. Although it coincides with two putatively annotated structural genes for cytosolic isoforms of PGM (At1g70730 and At1g70820) [25], no eQTL was detected at this position for variation in transcript levels of At1g70730, and only a minor eQTL was detected for transcript level variation of At1g70820 with an opposite additive effect to the activity QTL. This indicates that this activity QTL is due to genetic variation that acts *in cis *downstream of the transcript level, for example, a change in the stability or specific activity of this PGM isoform.

PGM catalyzes the reversible interconversion of G1P and G6P. During photosynthesis this reaction operates in the direction of G1P formation, and is required in the cytosol for the synthesis of sucrose and in the plastid for the synthesis of starch. The levels of the substrate and product of PGM were not affected by the PGM-activity QTLs (Figure 5b). Although minor QTLs were detected for G1P and G6P content, these did not co-locate with QTLs for PGM activity, suggesting that the size of the hexose phosphate pool varies independently of flux rates, as catalyzed by PGM. However, it should also be noted that the pools of G1P and G6P are larger in the cytosol than the plastid [48,49]. As the strong PGM-activity QTL is due to the plastidic PGM, subcellular resolution might be needed to detect changes in the plastid pools that are masked by the larger pools in the cytosol.

**Figure 6:**
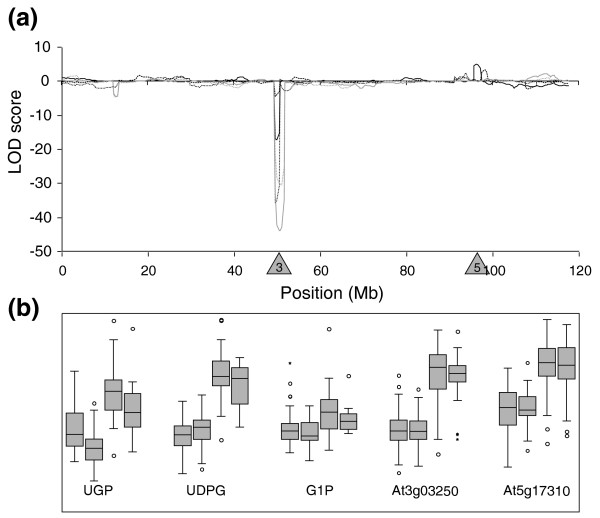
QTL profiles and boxplots of UGP related traits. **(a) **LOD scores plotted against genomic position; the sign of the LOD score is determined by the direction of effect (+, L*er *> Cvi; -, L*er *< Cvi). Black solid line, UGP activity; black dotted line, UDPG content; black dashed line, G1P content; gray solid line At3g03250 expression level; gray dotted line, At5g17310 expression level. Shaded triangles indicate positions of structural genes: 3, At3g03250; 5, At5g17310. **(b) **Boxplots for four genotypic classes. Each class represents genotypic identical individuals for the two QTLs at chromosomes 3 and 5 (from left to right: A_3_A_5_, A_3_B_5_, B_3_A_5_, B_3_B_5_; A = L*er*, B = Cvi). Boxplots show the median, interquartile range, outliers (circles) and extreme cases (asterisks) of individual variables. All traits are plotted in arbitrary units and ranged at similar scale by z-score standardization.

A different picture emerged for UGP. This enzyme converts the reversible conversion of G1P and uridine-triphosphate into UDPG and pyrophosphate. UGP is restricted to the cytosol and during photosynthesis operates in the direction of UDPG formation, which is then converted to sucrose. The relationship between the genetic regulation of the two genes was more complicated than for PGM, and there was a strong co-regulation between UGP activity and the levels of G1P and, to a lesser degree, UDPG (Figure 6).

Two QTLs with opposite effects were detected for UGP activity, each of them co-locating with a putatively annotated structural gene (Figure 6a). The UGP-activity QTL at the top of chromosome 3 co-locates with the structural gene At3g03250 and with an eQTL for this gene, which has the same direction of effect as the QTL for activity. This suggests that *cis*-regulated differences in transcript levels of At3g03250 explain the variation in UGP activity. The second QTL for UGP activity presents a more complicated picture. It maps to the upper arm of chromosome 5, and co-locates with the structural gene At5g17310. However, no eQTL co-located at this position; instead, a highly significant *trans*-acting eQTL for the *UGP *gene At5g17310 was detected at the same position as the chromosome 3 UGP-activity QTL and the At3g03250 eQTL, and with the same direction of effect. This implies that the UGP-activity QTL at chromosome 5 cannot be explained by transcription differences of At5g17310, but might instead result from *cis *polymorphisms in the coding sequence leading to a lower UGP protein stability or specific activity in the Cvi allele compared to the L*er *allele. Further, transcript level differences of At5g17310 might contribute to the chromosome 3 UGP-activity QTL. Thus, even though the encoded enzyme of the Cvi allele of At5g17310 might have a lower specific activity than the L*er *allele, it is much more strongly transcribed in lines carrying the Cvi genotype at the chromosome 3 locus (Figure 6b).

A strong QTL for both UDPG and G1P content was detected at the chromosome 3 locus (Figure 6a). Each metabolite showed the same direction of effect as the QTL for UGP activity and the eQTLs for gene transcript levels. The potential importance of the co-locating QTLs at the top of chromosome 3 for the At3g03250 and At5g17310 transcripts, UGPase activity, and the levels of UDPG and G1P is further indicated by the presence of a co-locating suggestive QTL for INV activity, and weak opposed QTLs for FK activity, and the levels of ChlA, ChlB, and amino acids (Figure 2). This was one of the few examples where several QTLs co-localized. Others included a co-location of a structural gene for INV and its eQTL, a strong QTL for INV activity and weak QTLs with the same direction of effect for glucose and fructose at the top of chromosome 1. The latter are the products of the INV reaction, which converts sucrose to reducing sugars. This set of QTLs is captured as PC3 in the principle components analysis (Tables 2 and 3).

## Discussion

### Natural variation in primary carbohydrate metabolism

Natural diversity provides a rich source of genetic perturbations. It has been effectively analyzed for carbohydrate metabolism by quantitative genetics in a number of studies in a variety of plant species [13,14,20,21,23,27,28,33,50-52]. However, most of these studies did not combine enzyme activity and metabolite level measurements, or incorporate transcription analysis of relevant genes. Here we present, for the first time, a comprehensive genetic analysis of all intermediate entities of the path from genotype to phenotype, including transcript levels, enzyme activities, and metabolite contents.

Our results reveal that there is extensive natural variation in primary carbohydrate metabolism in *Arabidopsis*. A substantial part of this variation was attributable to genetic regulation. In a relatively small RIL population, we were able to detect QTLs for most of the analyzed traits, including 15 QTLs for 10 of the 15 enzyme activities and 23 QTLs for 9 of the 11 metabolites analyzed in this study. Many of those QTLs could be explained by genetic variation in structural genes. Several other studies in *Arabidopsis *have also reported QTL analyses of carbohydrate metabolism traits in RIL populations [14,20-22,26,32]. There is reasonable agreement between our studies and these earlier investigations, both with respect to general features and the location of QTLs for specific traits.

The largest-scale study of enzyme activity QTLs to date was a study of ten enzymes, including PGI, PGM and FBP in a Columbia (Col) × L*er *RIL population [14]. No QTL was found for FBP, in contrast to our findings. For PGI two QTLs were found, but at positions other than the three loci identified in our study. A single QTL for PGM on chromosome 5 co-located with one of the QTLs identified in our study. PGM activity was previously analyzed in the L*er *× Cvi population [21] and at least three QTLs were reported, of which two co-locate with the two QTLs found in our study. In another study [20], INV activity was analyzed in the *Ler *× Cvi population revealing several QTLs, one of which is confirmed in our analyses.

With respect to metabolite QTLs, amino acid content was analyzed in the Bayreuth-0 (Bay-0) × Shahdara (Sha) population [32]. As in our study, a large number of QTLs was detected. While a few of these are confirmed in our study, co-location was not found for the most significant QTLs in these two studies. The extracts used in the aforementioned study [32] were also analyzed for starch, glucose, fructose, and sucrose content [26]. Multiple QTLs were detected for each analyzed trait under the two different environmental conditions. QTLs for starch content were not detected in our study, possibly due to differences in sampling time point and growth stage. Multiple QTLs for glucose, fructose, and sucrose were detected in our study, but co-location with QTLs reported in [26] was only observed for the strongest QTL for glucose content on chromosome 1 and for a minor QTL for fructose content on chromosome 3. QTLs for glucose, fructose, sucrose, G1P and G6P were also detected by [22] in the Col × Coimbra 24 (C24) RIL and near isogenic line populations but no co-location was found with QTLs in our study.

This lack of agreement might reflect genetic differences between the populations used and/or differences in developmental stage, timing of sampling, or environmental growth conditions. Earlier studies have shown that there are large differences in regulation of carbohydrate content when plants were grown under different nitrogen supply regimes [26,32]. Moreover, organ-specific regulation of enzyme activity has been reported [20,21]. These results illustrate that genetic regulation of primary carbohydrate metabolism is under spatial and temporal control involving a multitude of loci, which can be revealed depending on genotype, environment, development stages, and their mutual interactions.

There were large residual fractions of variance that could not be explained by detected QTLs, both in earlier studies and in our study. This might be due to sampling and analytical variation, and environmental and developmental differences between samples. It might also reflect the emerging picture that primary carbohydrate metabolism is subject to complex genetic regulation by many QTLs, and that many of these have a relatively small effect. Minor QTLs may fail to pass the QTL significance threshold. These minor small-effect QTLs in addition to extensive epistatic interactions may nevertheless collectively contribute to transgression and the large genetic variation that is observed. A genome-wide analysis of all marker-combinations provided evidence for multiple epistatic interactions for all traits and most of the genes that were investigated in the present study, indicating that epistasis contributes heavily to the observed genetic variation. Another indication of the complex regulation of primary carbohydrate metabolism is provided by the finding that specific QTLs were detected for most of the analyzed traits. In cases in which co-location of QTLs for different traits was observed, this might be due to the direct inter-dependence of the traits. For instance, UGP converts G1P into UDPG and all three traits map to a similar position on the genome. Another example is the co-location of QTLs for INV activity and glucose and fructose levels.

Despite the pattern of seemingly specific and independent regulation indicated by the position of the identified QTLs, there was a striking correlation pattern between many traits. Thus, positive correlations were observed between most enzyme activity levels, and between enzyme activities and the structural components, such as chlorophyll and protein contents. Weaker correlations were found between enzyme activities and some end products, such as sucrose, starch, and amino acids. Negative correlations were observed between enzyme activities and the more short-lived (see [49]) phosphorylated intermediates of carbohydrate metabolic pathways. These results suggest that, in addition to specific and independent regulation of metabolic pathways, a more general level of regulation is acting on carbohydrate metabolism.

This might be partly related to the growth and developmental status of the plant. To search for genetic factors that lead to broad changes in metabolic traits, we performed a principle component analysis. The first PC integrated changes in a large number of metabolic traits, and mapped to the position of *ERECTA *(At2g26330), a gene that is well known for its involvement in developmental control of *Arabidopsis*. This locus co-located with strong QTLs for protein content, ChlA, cytosolic PGI activity, and glucose content, and weaker QTLs for other metabolic traits. The *ERECTA *gene is polymorphic between the population's parental accessions L*er *and Cvi [42] and causal for many of the morphological and developmental differences observed between these accessions [53-55]. Moreover, *ERECTA *has been shown to exert pleiotropic effects on many growth related and metabolic traits [31,56,57] and was identified as a major hot-spot for *trans *eQTLs [41]. It is conceivable that *ERECTA *is responsible for a subtle simultaneous regulation of primary carbon metabolism, in parallel with, or as a consequence of, its effects on development. It was previously suggested that there may be such links, but without any specific suggestions as to which genes might be involved [13,18,22,23]. As stated in the introduction it is difficult to determine cause and consequence from correlation analyses but the observation that primary metabolism collectively and simultaneously differs in genotypes with known developmental dissimilarity favors a model in which control acts in the direction from development to metabolism.

### Relationship between structural gene expression and enzyme activity

Many metabolic conversions in plants are catalyzed by enzymes, and variation in enzymatic activity could have a high impact on metabolite levels and metabolic fluxes. It is conceivable that natural variation in enzyme activity could be generated by genomic variation in the structural genes encoding these enzymes, or by *trans*-acting regulatory mechanisms.

We found strong evidence that natural variation for enzyme activity levels is sometimes regulated *in cis *by variation in structural genes, and sometimes by *trans*-regulatory loci controlling the transcription of these genes. First, several examples were found for co-location of structural genes and enzyme activity QTLs (INV, PGM, cytosolic PGI, SPS, UGP), suggesting that natural variation for these genes is causal for the observed variation in enzyme activity. In some cases, *cis*-acting eQTLs were detected for these genes (INV, PGM, UGP), indicating that regulation is likely to occur on the transcriptional level. In other cases no *cis*-eQTLs were detected, indicating that regulation acts post-transcriptionally, possibly due to altered specific activity or protein stability. Secondly, examples were found for co-location of *trans*-acting eQTLs for structural genes and enzyme activity QTLs (cytosolic PGI), suggesting that *trans*-regulatory variation of these genes is causal for the observed variation in enzyme activity. Such regulation is likely to occur through transcriptional regulation of the structural gene due to variation for a distant regulator. Both *cis*- and *trans*-acting transcriptional as well as *cis*-acting post-transcriptional regulation of structural genes were identified as potential causes for observed variation in enzyme activity.

However, for many other enzymes the activity QTLs did not co-locate with structural genes or their eQTLs, suggesting that regulation occurs at multiple levels, and may be partly independent of variation in the (transcript levels of the) structural genes. Likewise, for many structural genes, eQTLs were detected that did not co-locate with QTLs for the encoded enzyme activity. Finally, for a number of structural genes no significant eQTL could be detected. This might be the result of low (variation in) transcript levels, which could not be detected in the microarray experiment.

Often we found only a weak to medium correlation between levels of enzyme activity and transcript levels of structural genes (Table 2). Apparently, variation observed in the transcript levels of these genes does not contribute to the variation observed in enzyme activity. There are many possible explanations for the lack of agreement between transcript levels and enzyme activities. It has already been noted that transcript levels often fail to correlate with enzyme activities across different environmental conditions [40,58,59]. This can be partly explained by the presence of multigene families. If several members are expressed at the same time, changes in the activity of one may be masked by the activity contributed by the others. Nevertheless, different genes of a gene family might have different specific activities for the metabolic conversions under study, for which also natural variation might be present between accessions. In a segregating population this diversity of genetic variants and possible epistatic interactions between them can severely complicate correlation analyses. Correlations might be difficult to establish when relationships between transcript levels and protein levels are not linear, due to delays in protein formation and/or activation upon transcription. Finally, lack of correlation can be simply a result of non-functionality at the sampled developmental stage or due to a dilution effect when genes are only transcribed in specific cells or tissues. Negative correlations might be the result of negative feedback due to high transcription levels of redundant genes, or phase shifts in diurnal rhythms of transcription and translation [19,60]. It is also possible that in some individual cases the genes have been incorrectly annotated, and actually have a different function.

### Different modes of action in the genetic control of enzymatic activity

For many enzymes, natural variation was observed in their level of activity, which, in many cases, was related to the levels of metabolites, including the substrates and products of the analyzed enzymes. In several cases QTLs for enzyme activity co-located with structural genes encoding these enzymes or eQTLs for those genes. Evaluation of the correlation patterns and QTL profiles between gene expression, enzyme activity and metabolite content indicates that the underlying causal genetic regulation varies from case to case. Various modes of genetic control acting via different mechanisms seem to act to regulate carbohydrate metabolism.

For PGM, which was one of the enzymes with the highest variation in activity in this RIL population, most of the variation could be explained by genetic factors. Parallel analysis of enzyme activity and structural gene expression suggested that *cis*-regulatory variation in transcription of one of the structural genes (At5g51820) was causal for the major PGM activity QTL. Another enzyme activity QTL was detected at the position of a second *PGM *structural gene (At1g70820). In this case, the changes in activity could not be explained by changes in transcription, because the enzyme activity and transcript level of the structural gene changed in opposite directions. This indicates that polymorphisms in coding regions of At1g70820 contribute to the observed variation in enzyme activity. There are several alternative explanations why an eQTL and activity QTL have different signs. One is that a polymorphism in the structural gene is leading to increased activity or protein stability, which results in changes of metabolites that weakly repress the transcription of the structural gene (negative feedback). Another is that there are actually two *cis *polymorphisms, one affecting transcription and one affecting protein function, which interact to regulate the eventual level of enzyme activity. The L*er *allele, compared to the Cvi allele, then leads to lower transcript levels but the encoded enzyme shows higher activity. For At1g70730, functional polymorphisms in the coding sequence on their own could explain the observed variation in enzyme activity, since no genetically regulated variation in transcript levels was observed for this gene. There were no QTLs for the substrates and products of the reaction at either of these sites. Thus, even though significant negative correlations were observed between PGM activity and its substrate and product G1P and G6P, these correlations were not caused by any of the detected activity QTLs for PGM. This suggests that other levels of regulation are also active for which no genomic variation could be detected within the analyzed population.

In contrast, the analysis of variation in the activity of UGP, its substrate and product and transcript levels of its encoding structural genes suggested that *trans*-regulated transcription differences are the major cause of variation in enzyme activity. A strong activity QTL and eQTL for At3g03250 co-located with the structural gene for At3g03250. While this might indicate that *cis*-regulation is responsible for the changes in UGP activity, other aspects of the results indicate that the situation is less straightforward. In particular, there is a strong homology in sequence and function between At3g03250 and At5g17310, which is the other member of the *UGP *gene family. As a highly significant eQTL was detected for both genes at an identical position (that is, co-locating with At3g03250), it is likely that they are co-regulated by the same genetic factor. This could imply that At3g03250 is not *cis*-regulated, as suggested earlier, but, like At5g17310, is regulated *in trans *by a tightly linked locus on chromosome 3. Further, the strong positive correlation between UDPG and G1P levels and UGP activity suggests that changes in metabolites might be the driving force for this *trans*-acting regulation. Metabolite levels were measured in illuminated material, when fluxes would be in the direction of UDPG formation and sucrose synthesis. The direction of effect and the position of the highly significant UDPG QTL can be explained by product accumulation, as a result of higher rates of conversion of G1P to UDPG by UGP. However, the direction of the QTL for the substrate G1P is against expectations since increasing conversion rates would be expected to lead to a decrease of the substrate. Instead, we hypothesize that higher levels of G1P or a related metabolite triggers up-regulation of the expression of both UGP encoding genes, leading to higher enzyme activity and accumulation of UDPG. This would mean that plants are able to sense and respond to changes in the levels of UDPG, G1P or a related metabolite. This has been suggested and shown also for other sugars [61-64].

Although it remains speculative to identify genetic factor(s) that determine(s) the variation observed in UDPG accumulation, it is interesting to note that the inorganic phosphate status in *Arabidopsis *affects the transcription of UGP-encoding genes [65,66]. Moreover, SPS, which converts UDPG into sucrose-6-P, is inhibited by inorganic phosphate [67]. Natural variation for phosphate and phytate, the major source of inorganic phosphate in plants, has been observed in the L*er *× Cvi population and a common QTL explaining most of the variation co-locates with the QTL for UDPG content and UGP activity [68]. Furthermore, a QTL for the accumulation of the phosphorylated hexoses G1P and G6P was detected at this position, which might indicate that high levels of inorganic phosphate result in elevated levels of phosphorylated sugars. This raises the possibility that variation in phosphorus levels control the accumulation of G1P, which in turn triggers the expression of UGP-encoding structural genes, leading to higher activity of UGP. Another interesting possibility is that the changes in UDPG levels and UGP activity may be connected to changes in uridine nucleotide metabolism. UDPG is an intermediate in sucrose synthesis, and is also formed during sucrose degradation via sucrose synthase. The major part of the uridine nucleotide pool in plants is present as UDPG, and changes in the level of uridine nucleotides can alter fluxes in sucrose metabolism [10,69].

It must be noted here that we have interpreted co-location of structural genes or their eQTLs with enzyme activity and metabolite QTLs as a strong indication of the involvement of these genes in the genetic regulation of traits. However, we reserve the possibility that local *in trans *regulation might occasionally have caused such co-location. Given the strong interconnectivity of the primary metabolism network and the many genes involved, it is not unlikely that genes with downstream effects on metabolism co-locate with structural genes by chance. Likewise, other modes of *trans*-regulation, such as epigenetic control, should not be excluded *a priori*. This was demonstrated by the silencing of *PAI *genes by an unlinked homologous inverted *PAI *repeat [70].

## Conclusion

Primary carbohydrate metabolism in plants is highly variable and susceptible to many perturbations. However, the mechanisms of perturbation, perception and signal transduction leading to altered metabolic fluxes are largely unknown. The genetic analysis of natural variants for plant primary metabolism has been shown to be an effective means for elucidating regulatory steps in the biological information flow from gene-to-function. We have shown that regulation occurs at different levels and identified many genetic loci involved in the control of various components of plant primary metabolism. The integrative and multi-parallel analyses of gene expression, enzyme activity and metabolite accumulation has revealed connectivity between these different entities but many cases of independent regulation at each level were also observed. The results indicate that much of the natural variation in plant primary metabolism can be attributed to allelic differences in structural genes of catalytic enzymes. In addition, variation independent of structural genes could be explained by the identification of regulatory loci. We also provide indicative evidence for metabolic signaling as one of the driving forces for modulations in metabolic routes.

Our findings underline the need for integrative studies for a thorough understanding of the complex regulation of plant metabolism. Such studies will have large implications for classical breeding as well as metabolic engineering of agronomical important crops. Moreover, they improve our knowledge about general mechanisms of genetic regulation of quantitative traits.

## Materials and methods

### Plant material and tissue collection

Aerial parts of seedlings from the accessions L*er *and Cvi and a population of 160 recombinant inbred lines derived from a cross between these parents [31,42] were grown and collected as described previously [31]. In brief, seeds of lines were sown in Petri dishes on 1/2MS (Murashigi and Skoog salts) agar and placed in a cold room for seven days. Petri dishes were then transferred to a climate chamber and seedlings were collected after seven days. Growing conditions were 16 h light (30 W.m^-2^) at 20°C, 8 h dark at 15°C and 75% relative humidity. Harvesting started 7 h into the light period and all lines were harvested in random order within 2 h. Plant material was stored at -80°C until further processing. All analyses described in this study were performed on the same material.

### Linkage map construction and anchoring to the physical map

The genetic map was constructed from a subset of the markers available [71], as described earlier [43]. In total, 144 markers were used, with an average spacing of 3.5 cM. The largest distance between two markers was 10.8 cM. The genetic map was anchored to the physical map as described in [43], with an almost linear genome-wide relation of 4.1 cM per Mbp.

### Metabolite and enzyme measurements

Metabolites were extracted and analyzed as described previously; ChlA, ChlB, amino acids, protein, sucrose, glucose, and fructose [13]; starch, G1P, and G6P [72]; and UDPG [31]. Enzymes were extracted as described in [40].

SuSy was assayed by incubating crude extract or UDP-glucose standards for 40 minutes in a freshly prepared medium containing 0.1 M Hepes/KOH pH 7.5, 20 mM MgCl_2_, 0.05% Triton X100, 0 or 50 mM UDP; 100 mM sucrose. The reaction was stopped by the addition of an equal volume of 0.5 M HCl in 100 mM Tricine/KOH pH 9 buffer. After incubation for 10 minutes at room temperature, and neutralization with 0.5 M NaOH, the UDPG formed was determined by a cycling assay at 340 nm. An equal volume of a solution containing 0.2 mM PPi, 0.2 M Tricine/KOH pH 8, 2 U.ml^-1 ^glycerokinase, 4 mM MgCl_2_, 2 U.ml^-1 ^glycerol-3-P dehydrogenase (GDH), 5 U.ml^-1 ^glycerol-3-P oxidase (GPOX), 1 U.ml^-1 ^UGP, 1 mM NaF and 1.1 mM NADH was added to the sample. Glycerokinase was produced as in [40].

UGP was assayed by incubating crude extract or UTP standards for 20 minutes in a freshly prepared medium containing 0.1 M Tricine/KOH pH 8, 4 mM MgCl_2_, 1.5 mM NaF, 0.05% Triton X100, 0 or 2 mM PPi, 5 mM UDPG, 10 U.ml^-1 ^glycerokinase. The reaction was stopped by adding an equal volume of 0.5 M HCl/100 mM Tricine/KOH pH 9. After incubation at room temperature for 10 minutes and neutralization with 0.5 M NaOH, UTP contents were determined by a cycling assay at 340 nm. For that, an equal volume of a mix containing 0.2 M Tricine/KOH pH 8, 2 U.ml^-1 ^GDH, 5 U.ml^-1 ^GPOX, 1.5 mM NADH and 1 mM MgCl_2 _was added to the samples.

All other enzymes were analyzed by similar protocols described previously; Inv, AGP, FBP, G6PDH, PFK, PFP, SPS, GK, FK [40]; PGI [13]; PGM [73]; and Rubisco [74]. Samples were randomized during extraction and analysis, and two biological replicates were analyzed for each trait.

### Microarray analyses

Transcript levels of genes were analyzed on two-color DNA-microarrays and published previously [43]. Resulting ^2^log signal intensities were used for correlation analyses in this study and ^2^log ratios between co-hybridized RILs were used for QTL analyses.

### Statistical analyses

Variance components of replicated measurements of enzyme activities and metabolite levels were used to estimate broad sense heritability according to the formula:

*H*^2 ^= *V*_*G*_/(*V*_*G *_+ *V*_*E*_)

where *V*_*G *_is the among-genotype variance component and *V*_*E *_is the residual (error) variance component.

Heritability of gene expression within the RIL population was calculated by using the pooled variance of the parents as an estimate of the within line variance:

*H*^2^_*RIL *_= (*V*_*RIL *_- *V*_*P*_)/*V*_*RIL*_

where *V*_*RIL *_and *V*_*P *_are the variance among adjusted expression intensities in the segregants and the pooled variance within parental measurements, respectively.

Spearman rank correlations between traits were determined in Excel (Microsoft) for mean trait values as follows:

$$\begin{array}{cc} R_{jk}=\frac{n\left(n^{2}-1\right)-6\sum_{i=1}^{n}{\left(y_{ij}-y_{ik}\right)}^{2}-\frac{1}{2}\left(T_{j}+T_{k}\right)}{\sqrt{\left[n\left(n^{2}-1\right)-T_{j}\right]\left[n\left(n^{2}-1\right)-T_{k}\right]}}, & \;j,\;k=1,2,...,m \end{array}$$

where *n *is the number of observations, *y *is the rank of observations for variables *j *to *m*, and $T_{j}=\sum t_{j}\left(t_{j}^{2}-1\right)$, *t*_*j *_being the number of ties of a particular value of variable *j*, and the summation being over all tied values of variable *j *[75].

QTL analyses for gene transcript levels were performed as described in [43]. For QTL analyses of metabolite and enzyme traits the computer program MapQTL version 5.0 [76] was used to identify and locate QTLs linked to the molecular markers using multiple QTL mapping [77,78]. Logarithm of odds (LOD) statistics were calculated at 0.5 cM intervals. Tests of 1,000 permutations were used to obtain an estimate of the number of type 1 errors (false positives). The genome-wide LOD score, which 95% of the permutations did not exceed, ranged from 2.4-2.7. A LOD score of 3.0, to correct for multiple testing, was then used as the significance threshold to declare the presence of a QTL. In the multiple QTL mapping model the genetic effect (μ_B_-μ_A_) and percentage of explained variance was estimated for each QTL, and 2 Mbp support intervals were established as an approximately 95% confidence level [79]. Co-location of (e)QTLs was defined as an overlap in the 2 Mbp-support intervals.

Genomic positions of genes were inferred from the *Arabidopsis *information resource [4]. When physical positions of genes fell in the 2 Mbp support interval of (e)QTLs this was considered as co-location.

PC and box plot analyses were performed in SPSS 12.0 (SPSS Inc., Chicago, IL, USA). Epistatic interactions were determined by performing a complete pair wise search (*p *< 0.001, determined by Monte Carlo simulations) for conditional and co-adaptive epistatic interactions for each trait using the computer program EPISTAT [80].

## Abbreviations

AGP, ADP-glucose pyrophosphorylase; ChlA, chlorophyl A; ChlB, chlorophyl B; Col, Columbia; Cvi, Cape verde islands; eQTL, expression QTL; FBP, Fructose-1,6-bisphosphate phosphatase, cytosolic isoform; FK, fructokinase; Fru, fructose; G1P, α-D-glucose-1-phosphate; G6P, α-D-glucose-6-phosphate; G6PDH, glucose-6-phosphate 1-dehydrogenase; GK, glucokinase; Glu, glucose; INV, acid soluble invertase, vacuolar; L*er*, Landsberg *erecta*; LOD, logarithm of odds; PC, principal component; PFK, ATP dependent phosphofructokinase; PFP, pyrophosphate:fructose-6-phosphate 1-phosphotransferase; PGI, phosphoglucose isomerase, cytosolic and plastidial isoforms; PGM, phosphoglucomutase; QTL, quantitative trait locus; RIL, recombinant inbred line; Rubisco, Ribulose bisphosphate carboxylase/oxygenase; SPS, sucrose phosphate synthase; Suc, sucrose; SuSy, Sucrose synthase; UDPG, UDP-D-glucose; UGP, UDP-glucose pyrophosphorylase.

## Authors' contributions

JK designed and carried out the experiments and drafted the manuscript. RS participated in carrying out the experiments. YG participated in designing the experiments and developing the activity assays. MCS developed the SuSy assay. JF participated in the statistical analyses. MK, MS and DV conceived of the study and participated in its design and coordination. All authors read and approved the final manuscript.

## Additional data files

The following additional data are available. Additional data file 1 contains Table S1, which provides an overview of various statistics of structural genes in relation to their encoding enzymes, and Table S2, which lists significant epistatic interactions of all analyzed traits. Additional data file 2 contains Figure S1 depicting the genome-wide distribution of epistatic loci for all analyzed traits.

## Supplementary Material

Additional data file 1 Table S1: an overview of various statistics of structural genes in relation to their encoding enzymes. Table S2: significant epistatic interactions of all analyzed traits. Click here for file

Additional data file 2 Genome-wide distribution of epistatic loci for all analyzed traits. Click here for file
