# Supplementary material for: Integrative analyses of genetic variation in enzyme activities of primary carbohydrate metabolism reveal distinct modes of regulation in Arabidopsis thaliana
Source: Genome Biol. 2008 Aug 18;9(8):R129. doi: 10.1186/gb-2008-9-8-r129 (PMC2575519; doi:10.1186/gb-2008-9-8-r129)
Supplement: Additional data file 1 — Table S1: an overview of various statistics of structural genes in relation to their encoding enzymes. Table S2: significant epistatic interactions of all analyzed traits. [file gb-2008-9-8-r129-S1.doc]

**Supplemental Table S1:** Statistics of structural genes.

Columns represent respectively the encoded enzymes, the location of the enzyme activity QTLs (lined up with a putative structural gene where possible), the AGI gene codes of structural genes, the position of the structural genes on the chromosome indicated in the AGI code, the heritability of gene transcript levels, the spearman rank correlation coefficient between enzyme activity and gene transcript levels, the *P*-value of the correlation coefficient, the chromosome number and, in parentheses, the position in Mbp, the LOD score, and the direction of effect (+, L*er* > Cvi; -, L*er* < Cvi) of detected eQTLs. Genes and eQTL positions in boldface co-locate with QTLs detected for enzyme activity. R-values in boldface are significant at a Bonferroni corrected *P*<0.01. When more then one eQTL was detected, positions, LOD scores, and effects of the different eQTLs are separated by a semicolon. For each enzyme the heritability of the total family of encoding genes and its correlation with the respective enzyme activity, as determined in a cluster analysis, are also displayed. na, not analyzed; ns, not significant.

| Enzyme | Position of QTLs | Gene ID and Position  (Mbp) | | H^2^ | Activity vs Transcript  R P | | Position of eQTLs | LOD score | Effect |
| --- | --- | --- | --- | --- | --- | --- | --- | --- | --- |
| Inv | 1(4.1) | **at1g12240** | 4.15 | 0.61 | 0.19 | 1.8E-02 | **1(4.1)** | 6.4 | - |
|  |  | at1g62660 | 23.20 | 0.00 | -0.06 | 4.8E-01 | 1(7.9); 3(20.0) | 3.7; 3.3 | -; - |
|  |  | Family |  | 0.42 | 0.04 | 5.6E-01 |  |  |  |
| AGP |  | at1g27680 | 9.63 | 0.82 | -0.08 | 3.2E-01 | 1(10.2) | 3.0 | - |
|  |  | at1g05610 | 1.67 | 0.95 | -0.04 | 6.1E-01 | 1(28.8); 3(20.5) | 4.0; 3.5 | -; + |
|  |  | at1g74910 | 28.14 | 0.63 | **0.24** | **3.2E-03** | 1(22.3); 1(26.4) | 3.7; 4.0 | +; + |
|  |  | at2g04650 | 1.62 | 0.00 | 0.03 | 7.4E-01 | ns |  |  |
|  |  | at2g21590 | 9.25 | 0.84 | -0.02 | 8.5E-01 | ns |  |  |
|  |  | at3g03250 | 0.75 | 0.94 | **-0.26** | **1.1E-03** | 1(12.5); 3(1.4); 3(20.5) | 3.2; 15.1; 3.2 | -; -; - |
|  | 4(12.4) |  |  |  |  |  |  |  |  |
|  |  | at4g39210 | 18.26 | 0.88 | -0.20 | 1.4E-02 | 3(18.6) | 3.5 | - |
|  |  | at5g17310 | 5.70 | 0.86 | **-0.23** | **3.9E-03** | 3(4.1) | 9.6 | - |
|  |  | at5g19220 | 6.46 | 0.72 | **0.23** | **3.4E-03** | 5(8.1) | 6.2 | + |
|  |  | at5g48300 | 19.59 | 0.00 | 0.15 | 6.2E-02 | ns |  |  |
|  |  | Family |  | 0.23 | 0.02 | 7.1E-01 |  |  |  |
| FBP |  | at1g43670 | 16.47 | 0.79 | 0.01 | 9.1E-01 | 1(12.2) | 3.1 | - |
|  |  | at3g54050 | 20.03 | 0.44 | -0.15 | 7.2E-02 | ns |  |  |
|  | 5(14.0) |  |  |  |  |  |  |  |  |
|  |  | at5g64380 | 25.76 | 0.91 | 0.03 | 7.5E-01 | 5(22.4) | 4.3 | - |
|  |  | Family |  | 0.35 | -0.13 | 9.2E-02 |  |  |  |
| G6PDH |  | at1g09420 | 3.04 | 0.90 | -0.06 | 4.5E-01 | 1(3.1) | 4.8 | - |
|  |  | at1g24280 | 8.61 | 0.69 | **0.31** | **8.4E-05** | 2(6.9) | 3.1 | + |
|  |  | at3g27300 | 10.08 | 0.58 | 0.17 | 3.7E-02 | 4(0.3) | 3.5 | - |
|  |  | at5g13110 | 4.16 | 0.00 | -0.02 | 7.6E-01 | ns |  |  |
|  |  | at5g35790 | 13.97 | 0.70 | 0.12 | 1.3E-01 | 4(0.3); 4(13.9); 5(16.7) | 3.1; 3.2; 4.7 | -; -; - |
|  |  | at5g40760 | 16.33 | 0.38 | 0.06 | 4.9E-01 | 5(16.7) | 8.9 | + |
|  |  | Family |  | 0.66 | 0.08 | 2.3E-01 |  |  |  |
| PFK |  | at1g43766 | 16.55 | na | na |  | na |  |  |
|  |  | at1g59810 | 22.01 | na | na |  | na |  |  |
|  |  | at2g22480 | 9.55 | 0.72 | 0.13 | 1.1E-01 | 1(18.0); 2(18.3); 5(2.5) | 3.7; 4.9; 3.6 | +; +;- |
|  |  | at4g26270 | 13.30 | 0.82 | **0.25** | **1.4E-03** | 2(10.0); 2(11.2) | 3.1; 3.5 | +; + |
|  |  | at4g29220 | 14.40 | 0.90 | -0.08 | 3.4E-01 | ns |  |  |
|  |  | at5g03300 | 0.80 | 0.85 | 0.10 | 2.0E-01 | 5(0.8) | 21.7 | + |
|  |  | at5g47810 | 19.37 | 0.87 | 0.04 | 6.4E-01 | ns |  |  |
|  |  | at5g56630 | 22.94 | 0.78 | -0.01 | 9.0E-01 | ns |  |  |
|  |  | at5g61580 | 24.78 | 0.78 | 0.09 | 2.8E-01 | ns |  |  |
|  |  | Family |  | 0.32 | 0.03 | 6.8E-01 |  |  |  |
| PFP |  | at1g12000 | 4.05 | 0.95 | 0.01 | 9.3E-01 | ns |  |  |
|  |  | at1g20950 | 7.30 | 0.48 | 0.01 | 9.3E-01 | ns |  |  |
|  |  | at1g76550 | 28.73 | 0.67 | **0.35** | **9.5E-06** | ns |  |  |
|  |  | at2g05150 | 1.86 | na | na |  | na |  |  |
|  |  | at4g04040 | 1.94 | 0.84 | -0.15 | 6.3E-02 | 1(3.8) | 3.5 | + |
|  |  | at4g08876 | 5.68 | na | na |  | na |  |  |
|  |  | at4g32840 | 15.84 | 0.91 | **0.27** | **7.7E-04** | ns |  |  |
|  |  | Family |  | 0.53 | 0.08 | 3.3E-02 |  |  |  |
| PGM |  | at1g23190 | 8.22 | 0.43 | 0.11 | 1.7E-01 | ns |  |  |
|  | 1(26.9) | **at1g70730** | 26.67 | 0.66 | 0.04 | 6.3E-01 | ns |  |  |
|  | 1(26.9) | **at1g70820** | 26.71 | 0.73 | -0.13 | 1.1E-01 | **1(28.0)** | 5.6 | - |
|  |  | at5g17530 | 0.58 | na | na |  | na |  |  |
|  | 5(20.9) | **at5g51820** | 21.08 | 0.96 | **0.69** | **4.4E-23** | 5(1.7); **5(21.0)** | 7.4; 36.6 | +; - |
|  |  | Family |  | 0.79 | **0.22** | **4.8E-03** |  |  |  |
| PGI(Cyt) |  | at1g30560 | 10.82 | 0.63 | -0.02 | 8.4E-01 | 4(6.6); 4(10.6) | 3.4; 3.4 | +; + |
|  | 1(16.8) |  |  |  |  |  |  |  |  |
|  | 2(11.2) | at4g25220 | 12.92 | 0.35 | **0.32** | **4.6E-05** | **2(11.2)** | 3.1 | + |
|  | 5(17.2) | **at5g42740** | 17.15 | 0.91 | 0.19 | 1.6E-02 | ns |  |  |
|  |  | Family |  | 0.56 | 0.12 | 1.4E-01 |  |  |  |
| PGI(Pla) |  | at4g24620 | 12.71 | 0.85 | -0.17 | 3.0E-02 | ns |  |  |
|  | 5(16.7) |  |  |  |  |  |  |  |  |
| PGI(Tot) |  | at1g30560 | 10.82 | 0.63 | 0.01 | 9.3E-01 | 4(6.6); 4(10.6) | 3.4; 3.4 | +; + |
|  | 1(14.9) |  |  |  |  |  |  |  |  |
|  |  | at4g24620 | 12.71 | 0.85 | **-0.23** | **3.5E-03** | ns |  |  |
|  |  | at4g25220 | 12.92 | 0.35 | 0.15 | 5.8E-02 | 2(11.2) | 3.1 | + |
|  |  | at5g42740 | 17.15 | 0.91 | 0.17 | 3.1E-02 | ns |  |  |
|  |  | Family |  | 0.63 | 0.01 | 5.2E-01 |  |  |  |
| SPS |  | at1g04920 | 1.39 | 0.94 | 0.10 | 2.2E-01 | ns |  |  |
|  |  | at1g16570 | 5.67 | 0.42 | 0.12 | 1.3E-01 | ns |  |  |
|  |  | at4g10120 | 6.31 | 0.93 | -0.08 | 3.1E-01 | 4(6.2) | 7.0 | + |
|  |  | at5g11110 | 3.54 | 0.76 | 0.13 | 1.2E-01 | 5(3.7) | 4.5 | + |
|  | 5(7.0) | **at5g20280** | 6.84 | 0.91 | **0.23** | **4.2E-03** | **5(7.2)** | 9.2 | + |
|  |  | Family |  | 0.78 | 0.07 | 3.7E-01 |  |  |  |
| SuSy |  | at1g73370 | 27.59 | 0.78 | 0.16 | 4.0E-02 | 5(14.0) | 8.2 | - |
|  |  | at3g43190 | 15.19 | 0.00 | -0.07 | 4.0E-01 | ns |  |  |
|  |  | at4g02280 | 0.99 | 0.71 | 0.07 | 3.8E-01 | ns |  |  |
|  |  | at5g20830 | 7.05 | 0.80 | 0.10 | 2.1E-01 | ns |  |  |
|  |  | at5g37180 | 14.74 | 0.66 | 0.14 | 7.7E-02 | ns |  |  |
|  |  | at5g49190 | 19.96 | 0.77 | **0.27** | **5.5E-04** | ns |  |  |
|  |  | Family |  | 0.52 | 0.10 | 8.1E-02 |  |  |  |
| GK |  | at1g30660 | 10.88 | 0.93 | 0.18 | 2.5E-02 | ns |  |  |
|  |  | at1g47840 | 17.62 | 0.71 | 0.04 | 6.5E-01 | 1(16.8) | 3.8 | - |
|  |  | at1g50460 | 18.70 | 0.94 | 0.02 | 7.9E-01 | 1(18.0) | 17.0 | - |
|  |  | at2g19860 | 8.58 | 0.97 | 0.10 | 2.3E-01 | ns |  |  |
|  |  | at3g20040 | 6.99 | 0.54 | **0.22** | **6.3E-03** | ns |  |  |
|  |  | at4g29130 | 14.35 | 0.04 | 0.07 | 4.0E-01 | ns |  |  |
|  |  | at4g37840 | 17.79 | 0.51 | 0.19 | 2.0E-02 | ns |  |  |
|  |  | Family |  | 0.31 | 0.06 | 4.2E-01 |  |  |  |
| FK |  | at1g06020 | 1.82 | 0.77 | -0.09 | 2.7E-01 | ns |  |  |
|  |  | at1g06030 | 1.83 | 0.44 | 0.01 | 8.8E-01 | ns |  |  |
|  |  | at1g30660 | 10.88 | 0.93 | 0.17 | 3.1E-02 | ns |  |  |
|  |  | at1g47840 | 17.62 | 0.71 | -0.11 | 1.6E-01 | 1(16.8) | 3.8 | - |
|  |  | at1g50390 | 18.67 | na | na |  | na |  |  |
|  |  | at1g50460 | 18.70 | 0.94 | -0.09 | 2.9E-01 | 1(18.0) | 17.0 | - |
|  |  | at1g66430 | 24.78 | 0.67 | -0.11 | 1.6E-01 | 1(28.8); 2(16.8) | 3.4; 3.5 | -; - |
|  |  | at1g69200 | 26.02 | 0.82 | -0.07 | 4.0E-01 | ns |  |  |
|  |  | at2g19860 | 8.58 | 0.97 | -0.03 | 7.2E-01 | ns |  |  |
|  |  | at2g31390 | 13.39 | 0.63 | -0.15 | 7.2E-02 | 2(12.5) | 5.0 | - |
|  |  | at3g20040 | 6.99 | 0.54 | **0.22** | **5.3E-03** | ns |  |  |
|  |  | at3g54090 | 20.04 | 0.94 | **0.26** | **1.2E-03** | 3(11.0) | 3.3 | + |
|  |  | at3g59480 | 21.99 | 0.91 | 0.05 | 5.6E-01 | ns |  |  |
|  |  | at4g10260 | 6.37 | na | na |  | na |  |  |
|  |  | at4g29130 | 14.35 | 0.04 | 0.18 | 2.5E-02 | ns |  |  |
|  |  | at4g37840 | 17.79 | 0.51 | 0.19 | 1.7E-02 | ns |  |  |
|  | 5(16.6) |  |  |  |  |  |  |  |  |
|  |  | at5g51830 | 21.09 | 0.95 | **0.27** | **5.8E-04** | 5(21.0) | 26.4 | - |
|  |  | Family |  | 0.44 | 0.03 | 8.2E-01 |  |  |  |
| UGP | 3(0.8) | **at3g03250** | 0.75 | 0.94 | **0.41** | **1.3E-07** | 1(12.5); **3(1.4)** | 4.5; 43.9 | -; - |
|  | 5(5.2) | **at5g17310** | 5.70 | 0.86 | **0.42** | **7.1E-08** | 1(12.5); **3(1.9)** | 4.6; 30.3 | -; - |
|  |  | Family |  | 0.91 | **0.45** | **2.6E-07** |  |  |  |
| Rubisco |  | at1g34630 | 12.69 | 0.85 | 0.19 | 1.9E-02 | 1(13.4) | 3.0 | - |
|  |  | at1g67090 | 25.05 | 0.37 | -0.03 | 7.3E-01 | ns |  |  |
|  | 3(20.5) |  |  |  |  |  |  |  |  |
|  |  | at5g38410 | 15.39 | 0.75 | 0.02 | 8.4E-01 | ns |  |  |
|  |  | at5g38420 | 15.40 | na | na |  | na |  |  |
|  |  | at5g38430 | 15.40 | 0.86 | 0.05 | 5.2E-01 | ns |  |  |
|  |  | Family |  | 0.52 | 0.04 | 6.1E-01 |  |  |  |

**Supplemental Table S2:** Significant interactions of analyzed traits. The second and seventh column represent the names of the markers in the pair-wise analysis. The third and eighth column the chromosome number and the fourth and ninth column the position of the marker on the chromosome. The fifth and tenth column represent the log likelihood ratio (LLR) and the sixth and eleventh column the P-values for a single locus effect of marker 1 and marker 2 respectively. The twelfth and thirteenth column represent the log likelihood ratio and the P-value of the interaction between marker 1 and marker 2. P-values were established only for markers with interaction LLR-values > 6.0 and are estimated from Monte Carlo simulations using 10,000 permutations. Shaded fields indicate markers showing, in addition to epistatic interaction, also a main effect. Markers in bold face indicate co-location with QTLs found for the respective trait.

|  | Marker 1 | | | | |  | Marker 2 | | | | |  | Interaction | |
| --- | --- | --- | --- | --- | --- | --- | --- | --- | --- | --- | --- | --- | --- | --- |
| Trait | Name | Chr. | Mb | LLR | P-value |  | Name | Chr. | Mb | LLR | P-value |  | LLR | P-value |
| Inv | BH106L | 1 | 12.5 | 0.2 | 4.9E-01 |  | DF231C | 5 | 5.6 | 0.0 | 7.6E-01 |  | 12.9 | 0.0E+00 |
| Inv | BF221L | 2 | 8.5 | 1.3 | 1.1E-01 |  | HH143C | 5 | 23.4 | 0.7 | 2.4E-01 |  | 6.8 | 4.0E-04 |
| Inv | DF77C | 3 | 0.3 | 1.1 | 1.3E-01 |  | GD239L | 5 | 12.9 | 0.4 | 3.8E-01 |  | 7.0 | 4.0E-04 |
| Inv | BH342C | 4 | 18.5 | 0.4 | 3.8E-01 |  | BH144L | 5 | 1.7 | 0.3 | 4.0E-01 |  | 6.3 | 1.2E-03 |
| AGP | EC480C | 1 | 4.4 | 0.1 | 6.4E-01 |  | CH169C | 4 | 4.5 | 0.1 | 6.9E-01 |  | 6.4 | 5.0E-04 |
| AGP | HH360L | 1 | 29.7 | 0.0 | 8.4E-01 |  | nga151 | 5 | 4.7 | 3.1 | 1.1E-02 |  | 6.0 | 1.9E-03 |
| AGP | T2N18 | 2 | 15.6 | 0.6 | 2.4E-01 |  | CH322C | 3 | 2.9 | 2.1 | 3.7E-02 |  | 6.4 | 1.5E-03 |
| AGP | BH342C | 4 | 18.5 | 0.7 | 2.3E-01 |  | BH180C | 5 | 2.8 | 1.3 | 1.1E-01 |  | 6.9 | 5.0E-04 |
| FBP | CH200C | 1 | 19.3 | 0.1 | 6.0E-01 |  | DF65L | 3 | 20.5 | 0.7 | 2.3E-01 |  | 6.5 | 1.8E-03 |
| FBP | T2N18 | 2 | 15.6 | 0.1 | 6.0E-01 |  | GH226C | 3 | 7.4 | 0.4 | 3.6E-01 |  | 7.4 | 5.0E-04 |
| FBP | DF77C | 3 | 0.3 | 0.3 | 4.3E-01 |  | HH143C | 5 | 23.4 | 0.9 | 1.8E-01 |  | 6.3 | 1.9E-03 |
| FBP | BH109L | 3 | 22.9 | 0.5 | 3.0E-01 |  | FD207L | 5 | 0.3 | 0.0 | 9.7E-01 |  | 6.5 | 8.0E-04 |
| G6PDH | GD97L | 1 | 13.4 | 1.8 | 5.2E-02 |  | CD87L | 3 | 20.0 | 0.5 | 3.2E-01 |  | 7.4 | 1.0E-04 |
| G6PDH | DF260L | 1 | 20.2 | 0.0 | 9.9E-01 |  | CC266L | 3 | 4.9 | 1.4 | 8.8E-02 |  | 10.0 | 4.0E-04 |
| G6PDH | CH284C | 2 | 6.4 | 2.8 | 1.5E-02 |  | CH88L | 5 | 15.0 | 1.5 | 7.5E-02 |  | 6.7 | 1.3E-03 |
| G6PDH | GD460L | 2 | 10.9 | 1.5 | 8.8E-02 |  | HH90L | 3 | 23.3 | 0.3 | 4.6E-01 |  | 11.4 | 1.0E-04 |
| G6PDH | AD92L | 3 | 9.8 | 3.9 | 3.0E-03 |  | DF300C | 5 | 13.6 | 1.9 | 4.6E-02 |  | 10.8 | 1.0E-04 |
| G6PDH | BF134C | 3 | 11.5 | 3.3 | 7.6E-03 |  | EC306L | 4 | 5.6 | 0.0 | 9.6E-01 |  | 6.3 | 1.8E-03 |
| G6PDH | AD292L | 5 | 1.3 | 0.5 | 3.1E-01 |  | GD350L | 5 | 19.3 | 0.0 | 8.0E-01 |  | 8.0 | 5.0E-04 |
| PFK | CD173L | 1 | 26.9 | 0.0 | 8.5E-01 |  | HH480C | 5 | 9.1 | 0.0 | 7.8E-01 |  | 7.7 | 5.0E-04 |
| PFK | CH610C | 2 | 2.9 | 0.0 | 8.8E-01 |  | CD116L | 5 | 15.6 | 0.1 | 7.1E-01 |  | 6.3 | 2.0E-03 |
| PFP | CRY2 | 1 | 1.2 | 1.6 | 7.2E-02 |  | FD59C | 1 | 16.8 | 2.5 | 2.1E-02 |  | 6.8 | 1.2E-03 |
| PFP | GB112L | 1 | 12.2 | 2.3 | 2.8E-02 |  | CH70L | 4 | 12.5 | 1.1 | 1.3E-01 |  | 6.6 | 1.3E-03 |
| PGM | AXR1 | 1 | 1.5 | 0.5 | 3.3E-01 |  | CD116L | 5 | 15.6 | 8.6 | 0.0E+00 |  | 6.2 | 1.1E-03 |
| PGM | AD106L | 1 | 10.4 | 0.0 | 8.0E-01 |  | GA1 | 4 | 1.2 | 0.0 | 8.8E-01 |  | 7.7 | 2.0E-04 |
| PGM | GB112L | 1 | 12.2 | 0.2 | 5.0E-01 |  | EC198L | 5 | 2.5 | 3.3 | 9.8E-03 |  | 9.0 | 4.0E-04 |
| PGM | **FD90L** | **1** | **28.0** | **9.3** | **0.0E+00** |  | GD318C | 3 | 9.0 | 0.9 | 1.6E-01 |  | 6.6 | 7.0E-04 |
| PGM | GD318C | 3 | 9.0 | 0.9 | 1.8E-01 |  | DF184L | 5 | 6.5 | 0.1 | 6.3E-01 |  | 6.6 | 1.5E-03 |
| PGM | BH342C | 4 | 18.5 | 0.0 | 8.4E-01 |  | BH180C | 5 | 2.8 | 3.1 | 1.2E-02 |  | 9.0 | 2.0E-04 |
| PGICyt | GD86L | 1 | 6.5 | 1.1 | 1.5E-01 |  | GB120C | 3 | 1.4 | 0.2 | 5.4E-01 |  | 10.6 | 0.0E+00 |
| PGICyt | GH157L | 1 | 25.4 | 0.1 | 6.6E-01 |  | EC198L | 5 | 2.5 | 0.1 | 6.2E-01 |  | 6.1 | 3.4E-03 |
| PGICyt | MSAT222 | 2 | 19.6 | 0.1 | 7.2E-01 |  | DF77C | 3 | 0.3 | 0.1 | 6.6E-01 |  | 7.0 | 1.5E-03 |
| PGICyt | FD207L | 5 | 0.3 | 0.5 | 3.0E-01 |  | CC262C | 5 | 21.8 | 0.8 | 2.1E-01 |  | 6.8 | 7.0E-04 |
| PGIPla | PVV4 | 1 | 0.2 | 0.3 | 4.3E-01 |  | CH690C | 5 | 0.8 | 0.0 | 9.4E-01 |  | 6.7 | 6.0E-04 |
| PGIPla | AD121C | 1 | 10.2 | 0.0 | 7.7E-01 |  | CC318C | 1 | 26.4 | 0.0 | 8.4E-01 |  | 6.3 | 1.0E-03 |
| PGIPla | FD111L | 3 | 4.1 | 0.2 | 5.8E-01 |  | CC188L | 5 | 10.2 | 1.0 | 1.5E-01 |  | 14.2 | 0.0E+00 |
| PGITot | AD121C | 1 | 10.2 | 0.2 | 5.6E-01 |  | HH375L | 1 | 23.7 | 0.1 | 7.0E-01 |  | 8.2 | 3.0E-04 |
| PGITot | DF260L | 1 | 20.2 | 2.5 | 2.2E-02 |  | CH65C | 2 | 7.6 | 0.6 | 2.7E-01 |  | 6.9 | 8.0E-04 |
| PGITot | GH247L | 2 | 12.9 | 1.3 | 1.1E-01 |  | BH342C | 4 | 18.5 | 0.2 | 4.8E-01 |  | 7.2 | 1.3E-03 |
| PGITot | FD111L | 3 | 4.1 | 0.6 | 3.0E-01 |  | CC188L | 5 | 10.2 | 0.1 | 6.0E-01 |  | 11.9 | 1.0E-04 |
| PGITot | FD207L | 5 | 0.3 | 0.6 | 2.8E-01 |  | BF168L | 5 | 23.5 | 0.1 | 7.1E-01 |  | 6.2 | 1.8E-03 |
| SPS | PVV4 | 1 | 0.2 | 0.6 | 2.9E-01 |  | FD59C | 1 | 16.8 | 1.6 | 7.5E-02 |  | 6.5 | 8.0E-04 |
| SPS | BH342C | 4 | 18.5 | 0.4 | 3.9E-01 |  | CH690C | 5 | 0.8 | 2.6 | 1.8E-02 |  | 8.4 | 2.0E-04 |
| SuSy | CH160L | 1 | 7.9 | 0.4 | 3.6E-01 |  | DFR | 5 | 17.2 | 0.4 | 3.9E-01 |  | 6.9 | 1.3E-03 |
| SuSy | BH109L | 3 | 22.9 | 0.3 | 4.8E-01 |  | BH342C | 4 | 18.5 | 0.4 | 3.8E-01 |  | 6.3 | 8.0E-04 |
| SuSy | HH90L | 3 | 23.3 | 0.2 | 5.3E-01 |  | BH92L | 4 | 6.2 | 0.0 | 8.6E-01 |  | 8.4 | 1.0E-04 |
| GK | GB112L | 1 | 12.2 | 0.0 | 9.4E-01 |  | GB490C | 4 | 15.0 | 0.0 | 8.0E-01 |  | 9.1 | 1.0E-04 |
| GK | DF77C | 3 | 0.3 | 0.5 | 3.2E-01 |  | BH96L | 5 | 10.0 | 0.4 | 3.5E-01 |  | 6.7 | 1.9E-03 |
| FK | GB112L | 1 | 12.2 | 0.0 | 9.9E-01 |  | HH159C | 4 | 13.9 | 0.4 | 3.5E-01 |  | 12.0 | 0.0E+00 |
| FK | EC88C | 1 | 20.9 | 0.1 | 7.1E-01 |  | DF231C | 5 | 5.6 | 0.0 | 8.9E-01 |  | 7.5 | 4.0E-04 |
| FK | F4I1 | 2 | 18.3 | 0.0 | 9.2E-01 |  | SC5 | 4 | 9.2 | 0.9 | 1.7E-01 |  | 9.0 | 5.0E-04 |
| FK | BF134C | 3 | 11.5 | 1.0 | 1.6E-01 |  | GH121L | 5 | 8.1 | 0.8 | 2.2E-01 |  | 6.6 | 1.3E-03 |
| FK | EC198L | 5 | 2.5 | 0.2 | 5.7E-01 |  | EG205L | 5 | 26.8 | 4.2 | 2.9E-03 |  | 9.1 | 5.0E-04 |
| UGP | PVV4 | 1 | 0.2 | 0.1 | 6.4E-01 |  | FD59C | 1 | 16.8 | 0.1 | 7.3E-01 |  | 7.5 | 4.0E-04 |
| UGP | AXR1 | 1 | 1.5 | 0.0 | 8.5E-01 |  | GD239L | 5 | 12.9 | 0.2 | 5.5E-01 |  | 6.6 | 3.0E-04 |
| UGP | EC480C | 1 | 4.4 | 0.0 | 9.6E-01 |  | GH226C | 3 | 7.4 | 3.1 | 1.2E-02 |  | 9.0 | 2.0E-04 |
| UGP | CD108L | 1 | 13.9 | 0.0 | 8.5E-01 |  | AD156C | 2 | 0.3 | 1.9 | 5.2E-02 |  | 6.6 | 2.0E-04 |
| UGP | HH360L | 1 | 29.7 | 1.2 | 1.4E-01 |  | DF65L | 3 | 20.5 | 0.4 | 3.9E-01 |  | 6.3 | 1.4E-03 |
| UGP | DF140C | 2 | 15.5 | 0.1 | 7.2E-01 |  | CD800C | 3 | 11.0 | 0.0 | 8.5E-01 |  | 6.0 | 1.5E-03 |
| Rubiscoini | GH157L | 1 | 25.4 | 0.0 | 8.4E-01 |  | GH580L | 2 | 2.5 | 0.0 | 7.5E-01 |  | 6.1 | 5.0E-04 |
| Rubiscoini | FD90L | 1 | 28.0 | 0.1 | 6.5E-01 |  | GD318C | 3 | 9.0 | 0.1 | 6.1E-01 |  | 6.9 | 7.0E-04 |
| Rubiscoini | HH360L | 1 | 29.7 | 0.0 | 9.1E-01 |  | DF231C | 5 | 5.6 | 0.1 | 6.6E-01 |  | 6.1 | 7.0E-04 |
| Rubiscoini | CH284C | 2 | 6.4 | 0.1 | 7.2E-01 |  | BH96L | 5 | 10.0 | 0.5 | 3.0E-01 |  | 7.9 | 1.0E-04 |
| Rubiscoini | GB150L | 2 | 10.0 | 0.8 | 2.0E-01 |  | DF65L | 3 | 20.5 | 1.0 | 1.6E-01 |  | 6.7 | 5.0E-04 |
| Rubiscoini | GD136C | 3 | 14.5 | 0.9 | 1.9E-01 |  | GB102L | 5 | 22.4 | 0.2 | 5.6E-01 |  | 6.2 | 7.0E-04 |
| Rubiscomax | CH284C | 2 | 6.4 | 0.6 | 2.7E-01 |  | CC188L | 5 | 10.2 | 0.1 | 6.4E-01 |  | 6.1 | 1.3E-03 |
| Rubiscomax | T6A23 | 2 | 16.2 | 0.4 | 3.8E-01 |  | FD111L | 3 | 4.1 | 0.6 | 2.8E-01 |  | 6.5 | 5.0E-04 |
| Rubiscomax | BH342C | 4 | 18.5 | 0.3 | 4.1E-01 |  | AD292L | 5 | 1.3 | 1.1 | 1.3E-01 |  | 6.7 | 8.0E-04 |
| Rubiscomax | FD207L | 5 | 0.3 | 1.8 | 5.3E-02 |  | BF168L | 5 | 23.5 | 0.0 | 9.4E-01 |  | 7.4 | 4.0E-04 |
| Rubiscoratio | HH335C | 1 | 3.1 | 0.4 | 4.6E-01 |  | EC88C | 1 | 20.9 | 1.0 | 1.4E-01 |  | 6.2 | 8.8E-03 |
| Rubiscoratio | BH147L | 1 | 4.1 | 0.4 | 4.4E-01 |  | CH88L | 5 | 15.0 | 0.1 | 7.9E-01 |  | 7.2 | 5.2E-03 |
| Rubiscoratio | GD86L | 1 | 6.5 | 0.8 | 2.2E-01 |  | FD98C | 3 | 18.9 | 1.0 | 1.5E-01 |  | 6.1 | 9.9E-03 |
| Rubiscoratio | HH360L | 1 | 29.7 | 1.6 | 4.4E-02 |  | DF231C | 5 | 5.6 | 2.0 | 2.0E-02 |  | 9.4 | 1.2E-03 |
| Rubiscoratio | GD460L | 2 | 10.9 | 0.3 | 5.4E-01 |  | BH342C | 4 | 18.5 | 0.3 | 5.6E-01 |  | 6.8 | 6.2E-03 |
| Rubiscoratio | MSAT222 | 2 | 19.6 | 0.1 | 7.0E-01 |  | BH88C | 3 | 6.0 | 0.8 | 2.1E-01 |  | 10.5 | 4.0E-04 |
| Rubiscoratio | MSAT222 | 2 | 19.6 | 0.1 | 7.3E-01 |  | AD129L | 5 | 8.6 | 4.2 | 2.0E-04 |  | 8.2 | 1.4E-03 |
| Rubiscoratio | CC266L | 3 | 4.9 | 1.0 | 1.4E-01 |  | EG205L | 5 | 26.8 | 0.1 | 8.0E-01 |  | 8.8 | 3.9E-03 |
| Rubiscoratio | HH117C | 3 | 16.6 | 0.0 | 9.9E-01 |  | EC198L | 5 | 2.5 | 0.3 | 4.5E-01 |  | 20.7 | 0.0E+00 |
| Rubiscoratio | BH109L | 3 | 22.9 | 0.2 | 6.3E-01 |  | FD167L | 4 | 11.4 | 0.2 | 5.5E-01 |  | 7.9 | 2.7E-03 |
| Rubiscoratio | ANL2 | 4 | 0.3 | 0.2 | 6.1E-01 |  | nga151 | 5 | 4.7 | 0.6 | 3.1E-01 |  | 6.7 | 4.6E-03 |
| Rubiscoratio | SC5 | 4 | 9.2 | 0.5 | 3.7E-01 |  | DF119L | 5 | 25.0 | 0.0 | 8.5E-01 |  | 7.4 | 3.1E-03 |
| Rubiscoratio | CH690C | 5 | 0.8 | 2.0 | 2.8E-02 |  | EG205L | 5 | 26.8 | 0.0 | 8.1E-01 |  | 8.7 | 3.6E-03 |
| chlA | EC66C | 1 | 6.0 | 3.1 | 1.3E-02 |  | CH169C | 4 | 4.5 | 0.1 | 6.6E-01 |  | 6.1 | 1.8E-03 |
| chlA | CD227C | 1 | 28.8 | 0.7 | 2.3E-01 |  | T7M24 | 4 | 2.6 | 0.2 | 5.0E-01 |  | 6.8 | 3.0E-04 |
| chlA | GH580L | 2 | 2.5 | 0.1 | 7.4E-01 |  | MSAT222 | 2 | 19.6 | 0.5 | 3.2E-01 |  | 6.4 | 7.0E-04 |
| chlA | CH284C | 2 | 6.4 | 0.1 | 6.9E-01 |  | DF300C | 5 | 13.6 | 0.4 | 3.6E-01 |  | 7.1 | 3.0E-04 |
| chlA | DF184L | 5 | 6.5 | 0.5 | 3.1E-01 |  | GD118C | 5 | 21.2 | 0.0 | 9.9E-01 |  | 7.7 | 4.0E-04 |
| chlB | AXR1 | 1 | 1.5 | 0.7 | 2.4E-01 |  | GH226C | 3 | 7.4 | 3.4 | 6.3E-03 |  | 7.1 | 6.0E-04 |
| chlB | GH580L | 2 | 2.5 | 0.0 | 9.2E-01 |  | MSAT222 | 2 | 19.6 | 0.0 | 8.5E-01 |  | 6.6 | 4.0E-04 |
| chlB | GH247L | 2 | 12.9 | 2.4 | 2.9E-02 |  | DF231C | 5 | 5.6 | 0.5 | 3.2E-01 |  | 6.9 | 3.0E-04 |
| Aa | EC66C | 1 | 6.0 | 3.1 | 2.9E-02 |  | FD111L | 3 | 4.1 | 0.3 | 5.8E-01 |  | 8.1 | 1.3E-03 |
| Aa | GD97L | 1 | 13.4 | 0.0 | 8.1E-01 |  | GB750C | 4 | 17.1 | 3.1 | 1.2E-02 |  | 7.9 | 4.0E-04 |
| Glc | SC5 | 4 | 9.2 | 0.0 | 9.0E-01 |  | EC151L | 5 | 16.7 | 2.3 | 3.2E-02 |  | 9.0 | 3.0E-04 |
| Fru | HH375L | 1 | 23.7 | 0.5 | 2.8E-01 |  | AD156C | 2 | 0.3 | 1.1 | 1.3E-01 |  | 7.3 | 2.0E-04 |
| Fru | GB120C | 3 | 1.4 | 4.5 | 2.6E-03 |  | BH342C | 4 | 18.5 | 0.2 | 5.8E-01 |  | 7.8 | 0.0E+00 |
| Fru | GH250C | 4 | 0.9 | 0.5 | 3.0E-01 |  | CH70L | 4 | 12.5 | 0.2 | 5.8E-01 |  | 7.7 | 6.0E-04 |
| Suc | AXR1 | 1 | 1.5 | 1.3 | 1.1E-01 |  | BH342C | 4 | 18.5 | 0.0 | 8.3E-01 |  | 6.5 | 1.0E-03 |
| Suc | HH335C | 1 | 3.1 | 1.2 | 1.2E-01 |  | GH226C | 3 | 7.4 | 0.2 | 5.6E-01 |  | 6.3 | 6.0E-04 |
| Suc | BF116C | 1 | 24.9 | 1.2 | 1.1E-01 |  | EC151L | 5 | 16.7 | 0.2 | 4.8E-01 |  | 7.7 | 3.0E-04 |
| Suc | DF77C | 3 | 0.3 | 0.1 | 6.9E-01 |  | GD296C | 3 | 17.6 | 0.6 | 2.8E-01 |  | 6.0 | 1.0E-03 |
| Suc | CC266L | 3 | 4.9 | 0.0 | 8.5E-01 |  | FD167L | 4 | 11.4 | 0.1 | 6.8E-01 |  | 7.1 | 2.0E-04 |
| Protein | AXR1 | 1 | 1.5 | 2.6 | 2.1E-02 |  | **GH226C** | **3** | **7.4** | **6.2** | **2.0E-04** |  | 6.0 | 2.1E-03 |
| Protein | AD121C | 1 | 10.2 | 3.1 | 1.5E-02 |  | HH375L | 1 | 23.7 | 0.0 | 8.5E-01 |  | 7.5 | 8.0E-04 |
| Protein | FD90L | 1 | 28.0 | 0.0 | 7.7E-01 |  | AD292L | 5 | 1.3 | 3.0 | 1.1E-02 |  | 12.6 | 0.0E+00 |
| Protein | **BH195L** | **2** | **12.5** | **2.7** | **2.1E-02** |  | DF65L | 3 | 20.5 | 2.1 | 4.4E-02 |  | 8.6 | 2.0E-04 |
| Protein | GH473C | 5 | 7.2 | 0.0 | 9.3E-01 |  | GD222C | 5 | 25.6 | 0.6 | 2.8E-01 |  | 6.1 | 1.5E-03 |
| Starch | HH375L | 1 | 23.7 | 0.0 | 7.6E-01 |  | BF221L | 2 | 8.5 | 0.0 | 8.6E-01 |  | 6.2 | 2.5E-03 |
| Starch | FD222L | 2 | 6.9 | 0.0 | 9.8E-01 |  | CD179L | 5 | 14.5 | 0.8 | 2.2E-01 |  | 6.6 | 1.0E-03 |
| Starch | EG75L | 3 | 2.5 | 1.1 | 1.3E-01 |  | DFR | 5 | 17.2 | 1.3 | 9.5E-02 |  | 6.7 | 1.6E-03 |
| Starch | CC266L | 3 | 4.9 | 0.0 | 9.0E-01 |  | FD154L | 4 | 6.6 | 0.8 | 2.1E-01 |  | 6.1 | 1.3E-03 |
| Starch | ANL2 | 4 | 0.3 | 1.4 | 9.8E-02 |  | BH342C | 4 | 18.5 | 0.2 | 5.1E-01 |  | 6.9 | 1.2E-03 |
| Starch | GH473C | 5 | 7.2 | 0.1 | 7.2E-01 |  | GD222C | 5 | 25.6 | 0.2 | 5.4E-01 |  | 6.8 | 6.0E-04 |
| UDPglucose | CRY2 | 1 | 1.2 | 1.6 | 6.8E-02 |  | BH109L | 3 | 22.9 | 0.0 | 8.7E-01 |  | 7.0 | 6.0E-04 |
| UDPglucose | BH147L | 1 | 4.1 | 3.1 | 1.1E-02 |  | GD239L | 5 | 12.9 | 0.1 | 6.0E-01 |  | 6.1 | 1.4E-03 |
| UDPglucose | EC66C | 1 | 6.0 | 3.3 | 7.7E-03 |  | CD173L | 1 | 26.9 | 0.3 | 4.6E-01 |  | 6.2 | 1.6E-03 |
| UDPglucose | GD86L | 1 | 6.5 | 4.4 | 1.5E-02 |  | EG75L | 3 | 2.5 | 37.0 | 0.0E+00 |  | 8.0 | 7.5E-03 |
| UDPglucose | HH360L | 1 | 29.7 | 0.3 | 4.3E-01 |  | **DF77C** | **3** | **0.3** | **49.3** | **0.0E+00** |  | 6.4 | 8.0E-04 |
| G1P | BH147L | 1 | 4.1 | 1.6 | 7.7E-02 |  | **DF77C** | **3** | **0.3** | **8.7** | **0.0E+00** |  | 6.4 | 2.8E-03 |
| G1P | g2395 | 1 | 7.3 | 0.5 | 3.1E-01 |  | GH121L | 5 | 8.1 | 2.0 | 4.3E-02 |  | 12.9 | 0.0E+00 |
| G1P | FD90L | 1 | 28.0 | 2.8 | 2.9E-02 |  | **GH473C** | **5** | **7.2** | **4.9** | **2.0E-03** |  | 6.8 | 2.8E-03 |
| G1P | HH360L | 1 | 29.7 | 0.7 | 2.4E-01 |  | DF65L | 3 | 20.5 | 1.4 | 8.8E-02 |  | 7.8 | 9.0E-04 |
| G1P | T6A23 | 2 | 16.2 | 0.3 | 4.1E-01 |  | CD329C | 4 | 10.6 | 0.1 | 6.1E-01 |  | 6.0 | 5.0E-03 |
| G1P | AD292L | 5 | 1.3 | 1.8 | 4.8E-02 |  | GD350L | 5 | 19.3 | 1.0 | 1.5E-01 |  | 10.5 | 1.0E-04 |
| G6P | HH335C | 1 | 3.1 | 0.2 | 4.9E-01 |  | HH480C | 5 | 9.1 | 1.4 | 9.5E-02 |  | 6.7 | 3.0E-04 |
| G6P | HH90L | 3 | 23.3 | 0.5 | 3.3E-01 |  | CD329C | 4 | 10.6 | 1.8 | 5.9E-02 |  | 8.9 | 1.0E-04 |
| At1g04920 | CH160L | 1 | 7.9 | 0.8 | 2.0E-01 |  | DF231C | 5 | 5.6 | 0.2 | 5.3E-01 |  | 6.8 | 4.0E-04 |
| At1g04920 | DF408C | 1 | 22.3 | 2.8 | 1.6E-02 |  | EC495C | 2 | 5.0 | 0.6 | 2.7E-01 |  | 6.5 | 6.0E-04 |
| At1g05610 | CRY2 | 1 | 1.2 | 0.0 | 9.1E-01 |  | GD222C | 5 | 25.6 | 0.6 | 2.8E-01 |  | 6.6 | 6.0E-04 |
| At1g06020 | g2395 | 1 | 7.3 | 1.0 | 1.7E-01 |  | BH88C | 3 | 6.0 | 0.1 | 6.6E-01 |  | 10.4 | 0.0E+00 |
| At1g06020 | T6A23 | 2 | 16.2 | 0.1 | 6.1E-01 |  | C6L9 | 4 | 1.7 | 0.0 | 8.9E-01 |  | 8.8 | 1.0E-04 |
| At1g06030 | EC480C | 1 | 4.4 | 4.9 | 1.7E-03 |  | T2N18 | 2 | 15.6 | 1.1 | 1.6E-01 |  | 6.7 | 7.0E-04 |
| At1g09420 | BF116C | 1 | 24.9 | 0.5 | 3.1E-01 |  | BH195L | 2 | 12.5 | 0.2 | 5.1E-01 |  | 7.0 | 1.0E-04 |
| At1g12000 | CD227C | 1 | 28.8 | 1.5 | 8.3E-02 |  | CD116L | 5 | 15.6 | 0.3 | 4.7E-01 |  | 8.8 | 0.0E+00 |
| At1g12000 | CH284C | 2 | 6.4 | 0.0 | 9.5E-01 |  | C6L9 | 4 | 1.7 | 1.4 | 9.0E-02 |  | 6.5 | 6.0E-04 |
| At1g12240 | DF408C | 1 | 22.3 | 0.1 | 6.0E-01 |  | DF184L | 5 | 6.5 | 0.3 | 4.6E-01 |  | 6.2 | 1.3E-03 |
| At1g12240 | BF116C | 1 | 24.9 | 0.0 | 8.7E-01 |  | EC495C | 2 | 5.0 | 0.0 | 8.9E-01 |  | 7.5 | 2.0E-04 |
| At1g12240 | GA1 | 4 | 1.2 | 0.0 | 8.2E-01 |  | EC151L | 5 | 16.7 | 2.5 | 2.2E-02 |  | 7.2 | 8.0E-04 |
| At1g16570 | EC66C | 1 | 6.0 | 0.1 | 6.4E-01 |  | CH88L | 5 | 15.0 | 0.0 | 7.9E-01 |  | 7.2 | 3.0E-04 |
| At1g16570 | GB150L | 2 | 10.0 | 0.0 | 8.1E-01 |  | CD179L | 5 | 14.5 | 0.1 | 7.0E-01 |  | 7.5 | 2.0E-04 |
| At1g16570 | GH247L | 2 | 12.9 | 0.1 | 7.5E-01 |  | HH171C | 3 | 21.2 | 0.2 | 5.5E-01 |  | 6.8 | 8.0E-04 |
| At1g16570 | GD136C | 3 | 14.5 | 4.0 | 4.6E-03 |  | GB102L | 5 | 22.4 | 0.0 | 8.8E-01 |  | 6.6 | 9.0E-04 |
| At1g16570 | BH342C | 4 | 18.5 | 0.3 | 4.6E-01 |  | CC262C | 5 | 21.8 | 0.2 | 5.5E-01 |  | 11.1 | 0.0E+00 |
| At1g16570 | CC188L | 5 | 10.2 | 0.3 | 4.4E-01 |  | EG205L | 5 | 26.8 | 0.8 | 2.1E-01 |  | 7.0 | 6.0E-04 |
| At1g20950 | BF116C | 1 | 24.9 | 2.6 | 1.8E-02 |  | EG205L | 5 | 26.8 | 0.0 | 8.2E-01 |  | 7.7 | 2.0E-04 |
| At1g20950 | FD222L | 2 | 6.9 | 0.1 | 7.3E-01 |  | BF148C | 3 | 10.6 | 0.1 | 7.5E-01 |  | 7.5 | 2.0E-04 |
| At1g23190 | EC66C | 1 | 6.0 | 0.7 | 2.4E-01 |  | CH215L | 1 | 23.9 | 2.4 | 2.9E-02 |  | 6.2 | 1.2E-03 |
| At1g24280 | PVV4 | 1 | 0.2 | 1.1 | 1.4E-01 |  | GH473C | 5 | 7.2 | 0.1 | 7.0E-01 |  | 7.1 | 3.0E-04 |
| At1g24280 | EC66C | 1 | 6.0 | 4.7 | 1.2E-03 |  | GB150L | 2 | 10.0 | 3.6 | 5.7E-03 |  | 6.5 | 7.0E-04 |
| At1g24280 | GB150L | 2 | 10.0 | 3.6 | 6.5E-03 |  | GH226C | 3 | 7.4 | 1.2 | 1.1E-01 |  | 6.6 | 3.0E-04 |
| At1g24280 | GD318C | 3 | 9.0 | 0.3 | 4.4E-01 |  | GH250C | 4 | 0.9 | 0.3 | 4.4E-01 |  | 9.3 | 0.0E+00 |
| At1g27680 | FD85C | 2 | 9.4 | 0.0 | 7.9E-01 |  | CD116L | 5 | 15.6 | 1.4 | 8.4E-02 |  | 6.4 | 6.0E-04 |
| At1g27680 | BF134C | 3 | 11.5 | 0.0 | 9.5E-01 |  | T7M24 | 4 | 2.6 | 0.2 | 5.5E-01 |  | 7.1 | 1.0E-04 |
| At1g27680 | FD207L | 5 | 0.3 | 1.8 | 5.7E-02 |  | GB102L | 5 | 22.4 | 2.7 | 2.1E-02 |  | 7.7 | 4.0E-04 |
| At1g30560 | AD156C | 2 | 0.3 | 0.1 | 6.9E-01 |  | BH96L | 5 | 10.0 | 0.0 | 7.8E-01 |  | 8.0 | 0.0E+00 |
| At1g30560 | Erecta | 2 | 11.2 | 0.9 | 1.8E-01 |  | **FD167L** | **4** | **11.4** | **6.1** | **4.0E-04** |  | 7.0 | 2.0E-04 |
| At1g30660 | BH106L | 1 | 12.5 | 0.8 | 2.1E-01 |  | CD179L | 5 | 14.5 | 0.9 | 1.7E-01 |  | 11.3 | 0.0E+00 |
| At1g34630 | BH88C | 3 | 6.0 | 1.2 | 1.2E-01 |  | EC151L | 5 | 16.7 | 0.0 | 7.5E-01 |  | 6.3 | 1.2E-03 |
| At1g34630 | HH440L | 3 | 15.1 | 2.4 | 2.2E-02 |  | BH342C | 4 | 18.5 | 0.4 | 4.0E-01 |  | 6.4 | 4.0E-04 |
| At1g47840 | AD121C | 1 | 10.2 | 1.0 | 1.6E-01 |  | BH109L | 3 | 22.9 | 2.2 | 3.5E-02 |  | 8.0 | 0.0E+00 |
| At1g50460 | GD86L | 1 | 6.5 | 0.4 | 3.7E-01 |  | GD318C | 3 | 9.0 | 0.4 | 3.5E-01 |  | 8.6 | 3.0E-04 |
| At1g50460 | DF408C | 1 | 22.3 | 15.0 | 0.0E+00 |  | CC266L | 3 | 4.9 | 0.0 | 9.7E-01 |  | 12.0 | 0.0E+00 |
| At1g50460 | GH157L | 1 | 25.4 | 3.3 | 1.0E-02 |  | AD156C | 2 | 0.3 | 0.2 | 5.5E-01 |  | 7.1 | 3.0E-04 |
| At1g50460 | BH195L | 2 | 12.5 | 2.5 | 2.7E-02 |  | DF300C | 5 | 13.6 | 0.2 | 5.8E-01 |  | 11.9 | 0.0E+00 |
| At1g62660 | GH580L | 2 | 2.5 | 0.3 | 4.8E-01 |  | HH143C | 5 | 23.4 | 6.5 | 4.0E-04 |  | 7.1 | 3.0E-04 |
| At1g67090 | GB112L | 1 | 12.2 | 1.7 | 5.7E-02 |  | CD179L | 5 | 14.5 | 1.6 | 6.7E-02 |  | 7.3 | 0.0E+00 |
| At1g67090 | DF231C | 5 | 5.6 | 0.1 | 6.8E-01 |  | CC262C | 5 | 21.8 | 0.1 | 7.4E-01 |  | 6.3 | 7.0E-04 |
| At1g69200 | g2395 | 1 | 7.3 | 0.0 | 9.0E-01 |  | CC188L | 5 | 10.2 | 0.3 | 4.2E-01 |  | 8.6 | 2.0E-04 |
| At1g69200 | DF140C | 2 | 15.5 | 0.7 | 2.4E-01 |  | FD154L | 4 | 6.6 | 1.0 | 1.5E-01 |  | 7.2 | 2.0E-04 |
| At1g69200 | EG75L | 3 | 2.5 | 1.6 | 6.4E-02 |  | SC5 | 4 | 9.2 | 2.3 | 2.8E-02 |  | 6.4 | 1.3E-03 |
| At1g69200 | GD318C | 3 | 9.0 | 0.2 | 5.3E-01 |  | BH81L | 5 | 19.4 | 1.4 | 9.6E-02 |  | 8.6 | 3.0E-04 |
| At1g69200 | CD329C | 4 | 10.6 | 2.3 | 2.5E-02 |  | AD292L | 5 | 1.3 | 0.8 | 2.1E-01 |  | 6.0 | 1.2E-03 |
| At1g70730 | FD59C | 1 | 16.8 | 2.7 | 2.0E-02 |  | FD98C | 3 | 18.9 | 0.0 | 8.6E-01 |  | 7.1 | 3.0E-04 |
| At1g70730 | BF116C | 1 | 24.9 | 1.3 | 1.0E-01 |  | HH143C | 5 | 23.4 | 1.0 | 1.6E-01 |  | 6.8 | 4.0E-04 |
| At1g70820 | HH335C | 1 | 3.1 | 2.0 | 4.8E-02 |  | EG75L | 3 | 2.5 | 1.0 | 1.7E-01 |  | 10.9 | 2.0E-04 |
| At1g70820 | BH106L | 1 | 12.5 | 1.4 | 9.0E-02 |  | MSAT222 | 2 | 19.6 | 0.0 | 9.1E-01 |  | 9.6 | 0.0E+00 |
| At1g70820 | HH360L | 1 | 29.7 | 7.1 | 1.1E-03 |  | EC198L | 5 | 2.5 | 0.1 | 8.0E-01 |  | 11.2 | 2.0E-04 |
| At1g70820 | DF140C | 2 | 15.5 | 0.3 | 4.1E-01 |  | BH342C | 4 | 18.5 | 1.8 | 5.9E-02 |  | 6.9 | 7.0E-04 |
| At1g70820 | AD92L | 3 | 9.8 | 0.8 | 2.2E-01 |  | EC198L | 5 | 2.5 | 0.1 | 7.5E-01 |  | 7.4 | 3.0E-04 |
| At1g74910 | PVV4 | 1 | 0.2 | 0.1 | 6.5E-01 |  | DF184L | 5 | 6.5 | 0.9 | 1.7E-01 |  | 8.2 | 1.0E-04 |
| At1g74910 | GD296C | 3 | 17.6 | 2.9 | 1.2E-02 |  | HH445L | 5 | 21.0 | 0.9 | 1.8E-01 |  | 6.1 | 9.0E-04 |
| At1g76550 | F4I1 | 2 | 18.3 | 0.0 | 9.6E-01 |  | HH159C | 4 | 13.9 | 0.0 | 8.3E-01 |  | 6.3 | 1.0E-03 |
| At2g04650 | HH171C | 3 | 21.2 | 0.4 | 3.4E-01 |  | BH144L | 5 | 1.7 | 1.3 | 1.1E-01 |  | 6.5 | 9.0E-04 |
| At2g04650 | EC306L | 4 | 5.6 | 1.6 | 7.2E-02 |  | GB235C | 5 | 14.0 | 1.4 | 9.8E-02 |  | 7.8 | 3.0E-04 |
| At2g19860 | MSAT222 | 2 | 19.6 | 0.0 | 7.8E-01 |  | FD207L | 5 | 0.3 | 0.2 | 5.5E-01 |  | 6.2 | 1.1E-03 |
| At2g19860 | FD98C | 3 | 18.9 | 0.8 | 2.1E-01 |  | AD292L | 5 | 1.3 | 1.2 | 1.2E-01 |  | 8.1 | 2.0E-04 |
| At2g19860 | FD207L | 5 | 0.3 | 0.2 | 5.6E-01 |  | GB235C | 5 | 14.0 | 0.1 | 6.9E-01 |  | 10.4 | 1.0E-04 |
| At2g21590 | PVV4 | 1 | 0.2 | 2.1 | 4.1E-02 |  | BH106L | 1 | 12.5 | 1.1 | 1.4E-01 |  | 6.2 | 1.1E-03 |
| At2g22480 | GH226C | 3 | 7.4 | 0.1 | 6.4E-01 |  | GA1 | 4 | 1.2 | 0.1 | 6.0E-01 |  | 9.0 | 0.0E+00 |
| At2g22480 | BF148C | 3 | 10.6 | 0.3 | 4.2E-01 |  | BH81L | 5 | 19.4 | 0.6 | 2.8E-01 |  | 7.1 | 1.0E-03 |
| At2g31390 | GB112L | 1 | 12.2 | 0.5 | 3.3E-01 |  | EC151L | 5 | 16.7 | 0.0 | 9.4E-01 |  | 7.1 | 2.0E-04 |
| At2g31390 | EG66L | 2 | 7.4 | 0.1 | 6.2E-01 |  | SC5 | 4 | 9.2 | 1.4 | 1.0E-01 |  | 6.9 | 5.0E-04 |
| At2g31390 | nga151 | 5 | 4.7 | 0.1 | 6.4E-01 |  | BH81L | 5 | 19.4 | 2.2 | 3.0E-02 |  | 6.5 | 5.0E-04 |
| At3g03250 | GD86L | 1 | 6.5 | 3.6 | 2.5E-02 |  | **EG75L** | **3** | **2.5** | **39.0** | **0.0E+00** |  | 8.6 | 5.0E-03 |
| At3g03250 | DF260L | 1 | 20.2 | 0.0 | 9.7E-01 |  | GB750C | 4 | 17.1 | 0.4 | 3.6E-01 |  | 6.4 | 3.0E-04 |
| At3g43190 | CRY2 | 1 | 1.2 | 1.9 | 5.0E-02 |  | CH322C | 3 | 2.9 | 0.1 | 6.5E-01 |  | 7.9 | 3.0E-04 |
| At3g43190 | BF128C | 3 | 15.8 | 0.4 | 3.7E-01 |  | FD167L | 4 | 11.4 | 0.5 | 3.4E-01 |  | 6.4 | 8.0E-04 |
| At3g43190 | EC306L | 4 | 5.6 | 0.0 | 8.6E-01 |  | EC198L | 5 | 2.5 | 0.3 | 4.7E-01 |  | 9.4 | 0.0E+00 |
| At3g43190 | GB750C | 4 | 17.1 | 0.0 | 9.9E-01 |  | DF119L | 5 | 25.0 | 0.3 | 4.7E-01 |  | 6.0 | 1.3E-03 |
| At3g54050 | FD90L | 1 | 28.0 | 1.4 | 9.0E-02 |  | GB235C | 5 | 14.0 | 0.0 | 8.8E-01 |  | 8.2 | 0.0E+00 |
| At3g54050 | EG66L | 2 | 7.4 | 0.6 | 2.6E-01 |  | C6L9 | 4 | 1.7 | 3.0 | 1.3E-02 |  | 6.5 | 7.0E-04 |
| At3g54050 | HH171C | 3 | 21.2 | 2.6 | 2.3E-02 |  | BH180C | 5 | 2.8 | 0.1 | 6.8E-01 |  | 6.8 | 1.0E-03 |
| At3g54090 | GD86L | 1 | 6.5 | 1.0 | 1.6E-01 |  | GD460L | 2 | 10.9 | 2.6 | 2.1E-02 |  | 6.4 | 3.0E-04 |
| At3g54090 | GD86L | 1 | 6.5 | 1.0 | 1.7E-01 |  | DF77C | 3 | 0.3 | 0.0 | 8.1E-01 |  | 8.8 | 0.0E+00 |
| At3g54090 | MSAT222 | 2 | 19.6 | 0.5 | 3.1E-01 |  | CC262C | 5 | 21.8 | 0.0 | 7.9E-01 |  | 7.5 | 0.0E+00 |
| At3g54090 | GH121L | 5 | 8.1 | 0.3 | 4.1E-01 |  | GD222C | 5 | 25.6 | 0.1 | 6.1E-01 |  | 6.5 | 5.0E-04 |
| At3g59480 | g2395 | 1 | 7.3 | 0.1 | 6.0E-01 |  | EC151L | 5 | 16.7 | 0.1 | 6.7E-01 |  | 6.2 | 6.0E-04 |
| At3g59480 | ANL2 | 4 | 0.3 | 0.9 | 1.8E-01 |  | GB750C | 4 | 17.1 | 0.0 | 8.8E-01 |  | 6.0 | 3.0E-04 |
| At4g02280 | EC88C | 1 | 20.9 | 0.5 | 3.0E-01 |  | GH226C | 3 | 7.4 | 0.6 | 2.8E-01 |  | 6.3 | 1.0E-03 |
| At4g02280 | BF325L | 2 | 1.0 | 0.0 | 9.9E-01 |  | BH96L | 5 | 10.0 | 0.1 | 6.8E-01 |  | 7.5 | 4.0E-04 |
| At4g04040 | PVV4 | 1 | 0.2 | 1.3 | 1.1E-01 |  | BH96L | 5 | 10.0 | 0.0 | 8.1E-01 |  | 7.0 | 2.0E-04 |
| At4g04040 | GH157L | 1 | 25.4 | 0.6 | 2.7E-01 |  | BH144L | 5 | 1.7 | 0.0 | 7.9E-01 |  | 6.8 | 5.0E-04 |
| At4g10120 | BH147L | 1 | 4.1 | 0.0 | 9.1E-01 |  | CH65C | 2 | 7.6 | 0.7 | 2.4E-01 |  | 7.9 | 3.0E-04 |
| At4g10120 | EC66C | 1 | 6.0 | 1.4 | 1.0E-01 |  | GB120C | 3 | 1.4 | 0.1 | 7.0E-01 |  | 7.9 | 3.0E-04 |
| At4g10120 | ANL2 | 4 | 0.3 | 1.2 | 1.2E-01 |  | CH70L | 4 | 12.5 | 1.8 | 5.2E-02 |  | 7.1 | 2.0E-04 |
| At4g24620 | CH284C | 2 | 6.4 | 0.8 | 2.0E-01 |  | GH250C | 4 | 0.9 | 0.1 | 7.1E-01 |  | 7.1 | 3.0E-04 |
| At4g25220 | HH360L | 1 | 29.7 | 0.2 | 5.6E-01 |  | GH121L | 5 | 8.1 | 0.0 | 7.6E-01 |  | 6.4 | 5.0E-04 |
| At4g25220 | CH610C | 2 | 2.9 | 0.5 | 3.4E-01 |  | HH171C | 3 | 21.2 | 0.8 | 2.0E-01 |  | 6.3 | 1.0E-03 |
| At4g25220 | GA1 | 4 | 1.2 | 1.3 | 9.8E-02 |  | GH473C | 5 | 7.2 | 0.0 | 8.5E-01 |  | 9.6 | 0.0E+00 |
| At4g26270 | GD97L | 1 | 13.4 | 0.1 | 6.3E-01 |  | CD87L | 3 | 20.0 | 2.3 | 3.2E-02 |  | 6.4 | 5.0E-04 |
| At4g26270 | EG357C | 2 | 16.8 | 0.9 | 1.7E-01 |  | AD112L | 3 | 22.4 | 1.2 | 1.2E-01 |  | 6.1 | 6.0E-04 |
| At4g29130 | HH117C | 3 | 16.6 | 1.6 | 7.5E-02 |  | DF119L | 5 | 25.0 | 0.7 | 2.4E-01 |  | 6.2 | 7.0E-04 |
| At4g29130 | CD87L | 3 | 20.0 | 1.2 | 1.2E-01 |  | FD207L | 5 | 0.3 | 0.3 | 4.5E-01 |  | 6.4 | 4.0E-04 |
| At4g29220 | HH375L | 1 | 23.7 | 1.0 | 1.6E-01 |  | BH120L | 2 | 13.9 | 0.1 | 6.8E-01 |  | 6.3 | 8.0E-04 |
| At4g32840 | CH215L | 1 | 23.9 | 0.0 | 9.5E-01 |  | EG66L | 2 | 7.4 | 0.5 | 3.4E-01 |  | 8.7 | 0.0E+00 |
| At4g37840 | AXR1 | 1 | 1.5 | 3.5 | 1.1E-02 |  | CH322C | 3 | 2.9 | 0.2 | 5.5E-01 |  | 7.0 | 4.0E-04 |
| At4g37840 | CH70L | 4 | 12.5 | 0.4 | 3.4E-01 |  | CD179L | 5 | 14.5 | 0.2 | 4.8E-01 |  | 6.0 | 7.0E-04 |
| At4g39210 | DF162L | 1 | 3.8 | 1.2 | 1.3E-01 |  | CH322C | 3 | 2.9 | 0.5 | 3.2E-01 |  | 6.1 | 1.1E-03 |
| At4g39210 | MSAT222 | 2 | 19.6 | 0.2 | 5.2E-01 |  | DFR | 5 | 17.2 | 1.4 | 8.9E-02 |  | 6.3 | 6.0E-04 |
| At5g03300 | FD90L | 1 | 28.0 | 2.9 | 2.2E-02 |  | **CH690C** | **5** | **0.8** | **58.3** | **0.0E+00** |  | 11.6 | 1.0E-04 |
| At5g03300 | BF325L | 2 | 1.0 | 5.6 | 1.6E-03 |  | **CH690C** | **5** | **0.8** | **58.3** | **0.0E+00** |  | 6.8 | 1.2E-03 |
| At5g03300 | GB150L | 2 | 10.0 | 0.9 | 1.8E-01 |  | DF328C | 3 | 18.6 | 0.5 | 3.3E-01 |  | 6.0 | 1.1E-03 |
| At5g03300 | HH171C | 3 | 21.2 | 2.3 | 4.1E-02 |  | **CH690C** | **5** | **0.8** | **58.3** | **0.0E+00** |  | 10.0 | 2.0E-04 |
| At5g03300 | VPMH47 | 4 | 15.7 | 5.9 | 2.2E-03 |  | **CH690C** | **5** | **0.8** | **58.3** | **0.0E+00** |  | 13.2 | 2.0E-04 |
| At5g11110 | CH610C | 2 | 2.9 | 1.6 | 7.1E-02 |  | EC151L | 5 | 16.7 | 3.4 | 7.0E-03 |  | 9.6 | 0.0E+00 |
| At5g13110 | FD90L | 1 | 28.0 | 0.0 | 7.9E-01 |  | DF140C | 2 | 15.5 | 0.2 | 5.8E-01 |  | 9.5 | 0.0E+00 |
| At5g17310 | EC66C | 1 | 6.0 | 2.5 | 4.3E-02 |  | **EG75L** | **3** | **2.5** | **32.9** | **0.0E+00** |  | 6.8 | 1.5E-03 |
| At5g17310 | GD86L | 1 | 6.5 | 2.6 | 2.0E-02 |  | BH342C | 4 | 18.5 | 0.2 | 5.0E-01 |  | 6.3 | 2.0E-04 |
| At5g17310 | CD108L | 1 | 13.9 | 2.5 | 2.2E-02 |  | CD87L | 3 | 20.0 | 1.0 | 1.5E-01 |  | 6.0 | 1.4E-03 |
| At5g17310 | CH610C | 2 | 2.9 | 0.0 | 7.6E-01 |  | DF77C | 3 | 0.3 | 32.8 | 0.0E+00 |  | 6.3 | 1.4E-03 |
| At5g19220 | GD86L | 1 | 6.5 | 2.3 | 4.9E-02 |  | CC266L | 3 | 4.9 | 0.1 | 7.7E-01 |  | 8.7 | 2.4E-03 |
| At5g19220 | CC318C | 1 | 26.4 | 1.0 | 1.5E-01 |  | CH88L | 5 | 15.0 | 3.0 | 1.2E-02 |  | 7.9 | 6.0E-04 |
| At5g19220 | GD296C | 3 | 17.6 | 0.0 | 9.3E-01 |  | HH445L | 5 | 21.0 | 0.2 | 5.5E-01 |  | 9.0 | 1.0E-04 |
| At5g19220 | DF65L | 3 | 20.5 | 1.1 | 1.2E-01 |  | CD329C | 4 | 10.6 | 0.0 | 8.9E-01 |  | 6.1 | 1.2E-03 |
| At5g20280 | BF325L | 2 | 1.0 | 0.0 | 8.4E-01 |  | BF168L | 5 | 23.5 | 2.7 | 1.9E-02 |  | 7.9 | 2.0E-04 |
| At5g20830 | BH147L | 1 | 4.1 | 0.9 | 1.9E-01 |  | EG205L | 5 | 26.8 | 0.5 | 3.4E-01 |  | 8.3 | 1.0E-04 |
| At5g20830 | DF77C | 3 | 0.3 | 1.3 | 9.4E-02 |  | DF65L | 3 | 20.5 | 0.1 | 6.9E-01 |  | 6.8 | 9.0E-04 |
| At5g20830 | DF65L | 3 | 20.5 | 0.1 | 6.2E-01 |  | BH180C | 5 | 2.8 | 9.4 | 0.0E+00 |  | 10.2 | 0.0E+00 |
| At5g35790 | CRY2 | 1 | 1.2 | 0.0 | 7.9E-01 |  | BH109L | 3 | 22.9 | 0.1 | 6.7E-01 |  | 7.6 | 1.0E-04 |
| At5g35790 | CD730C | 4 | 4.4 | 1.5 | 7.6E-02 |  | GD222C | 5 | 25.6 | 0.0 | 8.5E-01 |  | 6.7 | 5.0E-04 |
| At5g38410 | CH215L | 1 | 23.9 | 0.2 | 5.0E-01 |  | FD222L | 2 | 6.9 | 0.5 | 3.0E-01 |  | 6.7 | 1.0E-03 |
| At5g38430 | FD59C | 1 | 16.8 | 1.1 | 1.3E-01 |  | CC266L | 3 | 4.9 | 0.8 | 2.1E-01 |  | 10.2 | 1.0E-04 |
| At5g40760 | BH106L | 1 | 12.5 | 0.0 | 9.5E-01 |  | AD92L | 3 | 9.8 | 0.4 | 3.5E-01 |  | 6.2 | 4.0E-04 |
| At5g40760 | FD59C | 1 | 16.8 | 1.5 | 8.3E-02 |  | DF231C | 5 | 5.6 | 0.2 | 5.5E-01 |  | 6.8 | 4.0E-04 |
| At5g42740 | F4I1 | 2 | 18.3 | 3.1 | 1.0E-02 |  | CD730C | 4 | 4.4 | 0.5 | 3.0E-01 |  | 7.0 | 5.0E-04 |
| At5g42740 | BF134C | 3 | 11.5 | 0.5 | 3.2E-01 |  | C6L9 | 4 | 1.7 | 0.6 | 2.7E-01 |  | 7.3 | 5.0E-04 |
| At5g47810 | FD59C | 1 | 16.8 | 2.1 | 3.8E-02 |  | CD730C | 4 | 4.4 | 0.0 | 8.3E-01 |  | 6.4 | 5.0E-04 |
| At5g49190 | BH106L | 1 | 12.5 | 0.5 | 3.3E-01 |  | BH92L | 4 | 6.2 | 1.2 | 1.2E-01 |  | 7.0 | 2.0E-04 |
| At5g49190 | BF206L | 1 | 18.0 | 0.0 | 9.5E-01 |  | HH480C | 5 | 9.1 | 0.0 | 8.0E-01 |  | 6.4 | 1.1E-03 |
| At5g51820 | GD86L | 1 | 6.5 | 0.0 | 8.3E-01 |  | **BH81L** | **5** | **19.4** | **34.1** | **0.0E+00** |  | 6.3 | 1.4E-03 |
| At5g51820 | GD97L | 1 | 13.4 | 1.2 | 1.2E-01 |  | **EC198L** | **5** | **2.5** | **6.9** | **1.0E-04** |  | 7.9 | 1.0E-04 |
| At5g51820 | GD318C | 3 | 9.0 | 1.8 | 5.6E-02 |  | DF184L | 5 | 6.5 | 0.1 | 6.8E-01 |  | 14.1 | 0.0E+00 |
| At5g51820 | HH117C | 3 | 16.6 | 0.2 | 5.4E-01 |  | **CC262C** | **5** | **21.8** | **31.9** | **0.0E+00** |  | 9.5 | 0.0E+00 |
| At5g51820 | VPMH19 | 4 | 16.9 | 1.1 | 1.4E-01 |  | GB102L | 5 | 22.4 | 26.1 | 0.0E+00 |  | 6.3 | 1.0E-03 |
| At5g51830 | GH226C | 3 | 7.4 | 0.1 | 6.6E-01 |  | GB102L | 5 | 22.4 | 33.6 | 0.0E+00 |  | 18.1 | 1.0E-04 |
| At5g51830 | GD318C | 3 | 9.0 | 0.5 | 3.1E-01 |  | DF184L | 5 | 6.5 | 0.9 | 1.8E-01 |  | 9.8 | 1.0E-04 |
| At5g51830 | DF328C | 3 | 18.6 | 0.6 | 2.5E-01 |  | nga151 | 5 | 4.7 | 0.1 | 7.0E-01 |  | 12.9 | 0.0E+00 |
| At5g51830 | CD87L | 3 | 20.0 | 1.4 | 1.0E-01 |  | **HH445L** | **5** | **21.0** | **62.9** | **0.0E+00** |  | 10.5 | 6.0E-04 |
| At5g51830 | FD207L | 5 | 0.3 | 1.6 | 7.4E-02 |  | GD350L | 5 | 19.3 | 42.5 | 0.0E+00 |  | 6.4 | 4.3E-03 |
| At5g61580 | GD97L | 1 | 13.4 | 0.2 | 5.0E-01 |  | GD350L | 5 | 19.3 | 0.4 | 3.6E-01 |  | 7.0 | 8.0E-04 |
| At5g64380 | GD97L | 1 | 13.4 | 1.9 | 4.9E-02 |  | VPMH47 | 4 | 15.7 | 0.0 | 8.6E-01 |  | 6.1 | 1.3E-03 |
| At5g64380 | FD90L | 1 | 28.0 | 0.5 | 3.0E-01 |  | HH159C | 4 | 13.9 | 0.1 | 7.0E-01 |  | 6.0 | 1.1E-03 |
| At5g64380 | BH109L | 3 | 22.9 | 0.6 | 2.9E-01 |  | EG205L | 5 | 26.8 | 3.4 | 8.6E-03 |  | 6.6 | 3.0E-04 |
| At5g64380 | BH109L | 3 | 22.9 | 0.6 | 3.0E-01 |  | EG205L | 5 | 26.8 | 3.4 | 7.9E-03 |  | 6.6 | 3.0E-04 |
