# Supplementary material for: Integrative analyses of genetic variation in enzyme activities of primary carbohydrate metabolism reveal distinct modes of regulation in Arabidopsis thaliana
Source: Genome Biol. 2008 Aug 18;9(8):R129. doi: 10.1186/gb-2008-9-8-r129 (PMC2575519; doi:10.1186/gb-2008-9-8-r129)
Supplement: Additional data file 2 — Genome-wide distribution of epistatic loci for all analyzed traits. [file gb-2008-9-8-r129-S2.pdf]

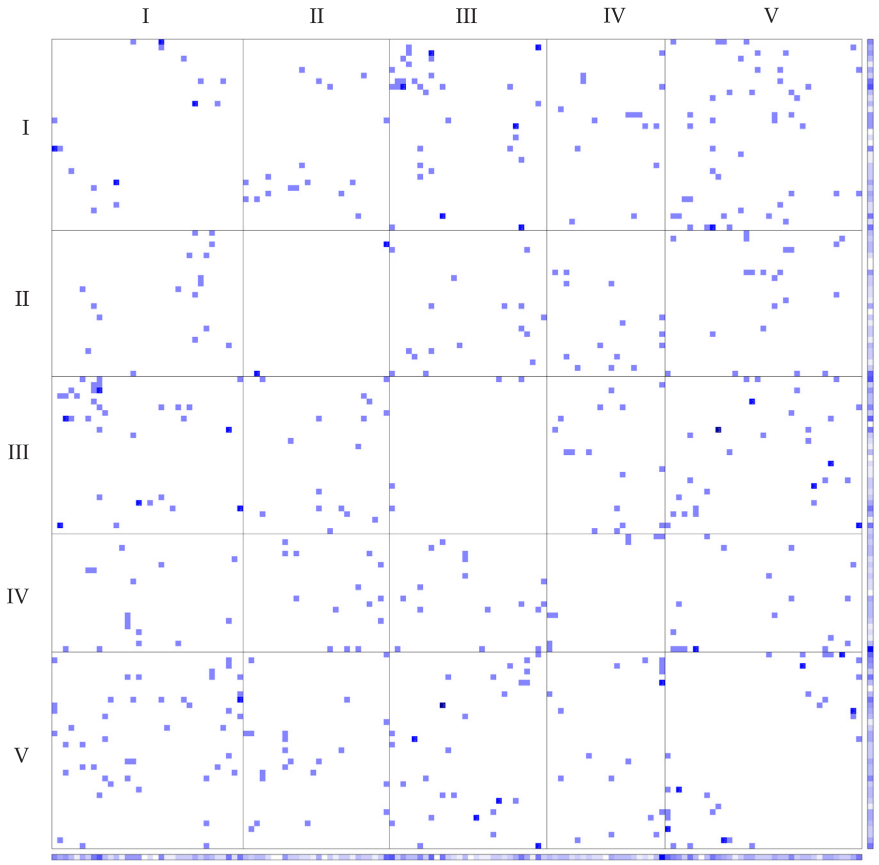

Figure S1. Genome-wide distribution of epistatic loci for all analyzed traits
Each pixel indicates an epistatic interaction marker pair. Only pairs with unlinked markers (separated by more than 50 cM) were tested for epistatic interactions. Vertical and horizontal lines depict chromosomal borders. Note that each interaction is depicted twice (mirror axis on the diagonal). The intensity bars at the bottom and on the right indicate the frequency of genomic positions involved in epistatic interactions.
